# Ligand driven heterolytic O–O bond cleavage in a non-haem phenolato-Fe(iii)–OOH complex to yield a formal Fe(v)=O intermediate

**DOI:** 10.1039/d5dt01477h

**Published:** 2025-09-08

**Authors:** Daniël R. Duijnstee, Marika Di Berto Mancini, C. Maurits de Roo, Duenpen Unjaroen, Moniek Tromp, Ronald Hage, Wesley R. Browne, Marcel Swart

**Affiliations:** a Stratingh Institute for Chemistry, Faculty of Science and Engineering, University of Groningen Nijenborgh 3 9747AG Groningen The Netherlands w.r.browne@rug.nl; b Zernike Institute for Advanced Materials, Faculty of Science and Engineering, University of Groningen Nijenborgh 3 9747AG Groningen The Netherlands; c IQCC & Dept. Química, Universitat de Girona Campus Montilivi 17003 Girona Spain; d ICREA Pg. Lluís Companys 23 08010 Barcelona Spain marcel.swart@udg.edu

## Abstract

Fe(v)=O species can be generated by the heterolytic cleavage of the O–O bond of corresponding Fe(iii)–OOH species. In haem complexes the redox non-innocence of the ligand facilitates such heterolytic cleavage, however non-haem iron complexes generally show homolytic cleavage to form an Fe(iv)=O species and a hydroxyl radical. The hydroxyl radical formed is undesirable due to its non-selective reactivity. Here we show that the redox non-innocence of a phenolato ligand moiety in the complex [LFe(iii)(μ-O)Fe(iii)L]^2+^, where L is 2-(((di(pyridin-2-yl)methyl)(pyridin-2-ylmethyl)amino)methyl)phenolate, facilitates heterolytic O–O bond cleavage, similar in manner to that observed with haem Fe(iii)–OOH species, to yield a formal Fe(v)=O intermediate. Although not observed directly, the intermediacy of an Fe(v)=O species is manifested in the immediate appearance of a doubly oxidised bis-phenolato bridged complex observed by time resolved UV/vis absorption and resonance Raman spectroscopy. This complex is formed by C–C coupling at the *para* position of the phenolato moiety of the ligand. The pathways to form the final complex *via* various Fe(iv)=O and Fe(v)=O intermediates are investigated by DFT methods, which indicate that the impact of the phenolato moiety is due to its redox non-innocence primarily. The ability of the phenolato moiety to transfer charge and spin density induces a switch in the mechanism of O–O bond cleavage from homolytic to heterolytic manifested in the radical character at the *para*-position needed for C–C bond formation and the high oxidation state of the first observed product.

## Abstract 2

Observation of phenoxyl radical coupled products confirm the generation of an Fe(v)=O species by the heterolytic cleavage of the O–O bond of corresponding Fe(iii)–OOH species.

## Graphical abstract

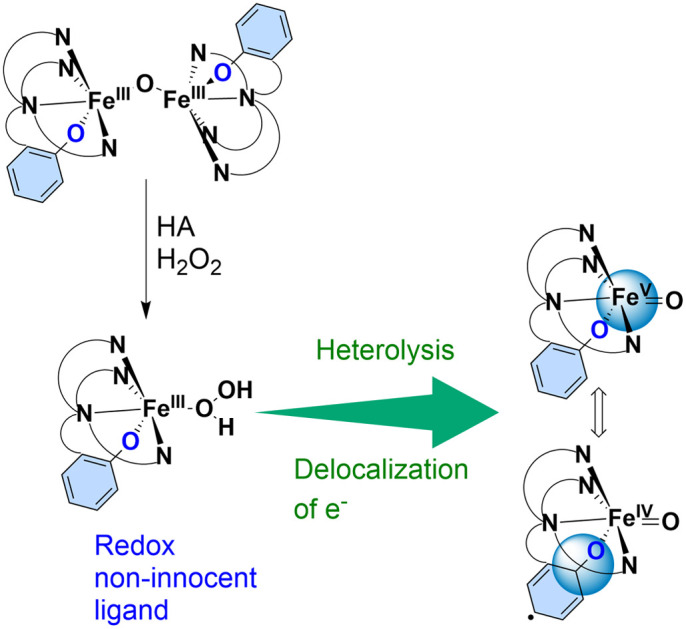

## Introduction

High valent iron oxido species (Fe(iv)=O and Fe(v)=O) are important intermediates in many of the enzymes responsible for oxidative organic transformations.^1–4^ Biomimetic and bio-inspired synthetic complexes, from which Fe(iv)=O species can be generated, have been reported over the last two decades.^2,5–9^ The potency of these Fe(iv)=O species has increased over time through ligand design strategies aimed at controlling reactivity primarily.^10,11^ Ultimately, the goal of these studies is to understand how natural systems such as TauD^12^ and methane monoxygenase,^13^ for example, can be sufficiently reactive to break strong C–H bonds.^14^ Of particular interest in regard to oxidation catalysis is the formation of these species with H_2_O_2_ as terminal oxidant, *e.g.*, through homolytic cleavage of the O–O bond of an Fe(iii)–OOH intermediate; however this pathway generates one equivalent of hydroxyl radical also. Accessing the Fe(v)=O state through O–O bond heterolysis in an Fe(iii)–OOH species is of interest to avoid hydroxyl radical formation,^15–17^ and to increase reactivity towards organic substrates further. Indeed the formation of hydroxyl radicals, *i.e.* through Fe(iii)–(OOH) is highly undesirable, especially in biological systems, where the hydroxyl radical is one of the most damaging of the reactive oxygen species (ROS).

**Homolytic pathways**:

1 LFe(iii)–OOH → LFe(iv)=O + HO˙

2 LFe(iii)–(HOOH) → LFe(iv)=O + H^+^ + HO˙

**Heterolytic pathway**:

3 LFe(iii)–(HOOH) → LFe(v)=O + H_2_O

**Fig. 1:**
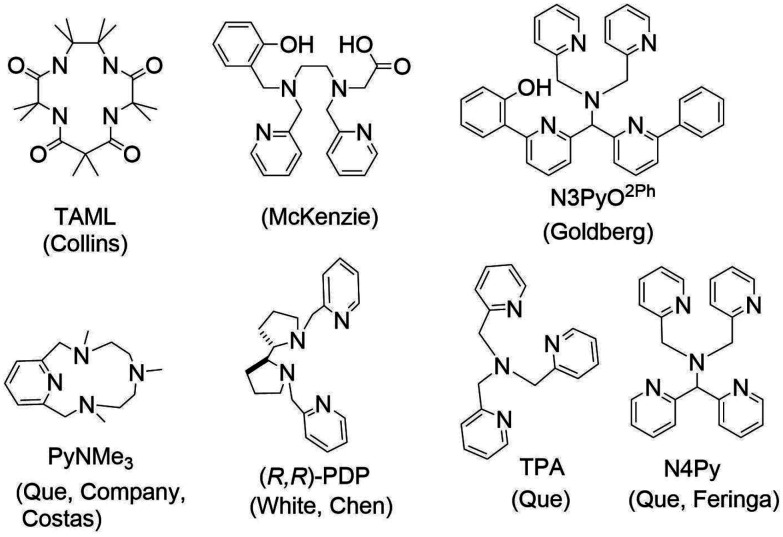
Ligands for iron complexes discussed in the text.

Fe(v)=O species are relatively rare in synthetic non-haem systems, with only a few examples to date. The N4 coordinated Fe-TAML family of complexes, developed originally by Collins and coworkers, stands out in that the Fe(iv)=O and Fe(v)=O complexes are sufficiently stable to allow for spectroscopic characterization. The higher oxidation states are stabilised by the electron rich deprotonated amide ligands (Fig. 1). More recently, Que, Costas and coworkers^18–21^ have proposed the transient formation of Fe(v)=O species with non-haem N4 (pyridyl) coordinated iron complexes (PyNMe_3_,^22^ (*R*,*R*)-PDP^23^ and TPA, see Fig. 1 for ligand structures) with peroxy acids. The two (*cis*) coordination sites available allow bidentate coordination of peracids, facilitating heterolytic O–O bond cleavage in Fe(iii)–peroxy acid complexes.^24–29^ The generation of Fe(v)=O species through heterolysis of the O–O bond has been inferred for synthetic non-haem Fe(iii)–OOH complexes by Que, Costas, Nordlander, McKenzie and others.^15–17,30–32^ McKenzie and coworkers reported N_4_O (carboxylate containing) ligands (Fig. 1) that provide a first coordination sphere with H_2_O_2_ similar to that with N_4_ ligands and peroxy acids.^30^ The products formed upon reaction of the (N_4_O)Fe(iii) complex with H_2_O_2_ indicated the intermediacy of an Fe(v)=O species. These earlier reports indicate that ligand design can support O–O bond heterolysis.

Indeed in nature, the ligand environment is essential for driving metal centred reactions by expanding the range of formal redox states beyond those typically available with first row transition metals.^33–37^ The redox non-innocence of ligands^38–45^ is essential to the activity of these enzymes.^46–48^ In the haem peroxidases, for example, coordination of H_2_O_2_ to the Fe(iii) haem centre is followed by O–O bond heterolysis yielding a formal Fe(v)=O state.^46,49^ This species is better described as an Fe(iv)=O with a one electron oxidized haem ligand.

**Fig. 2:**
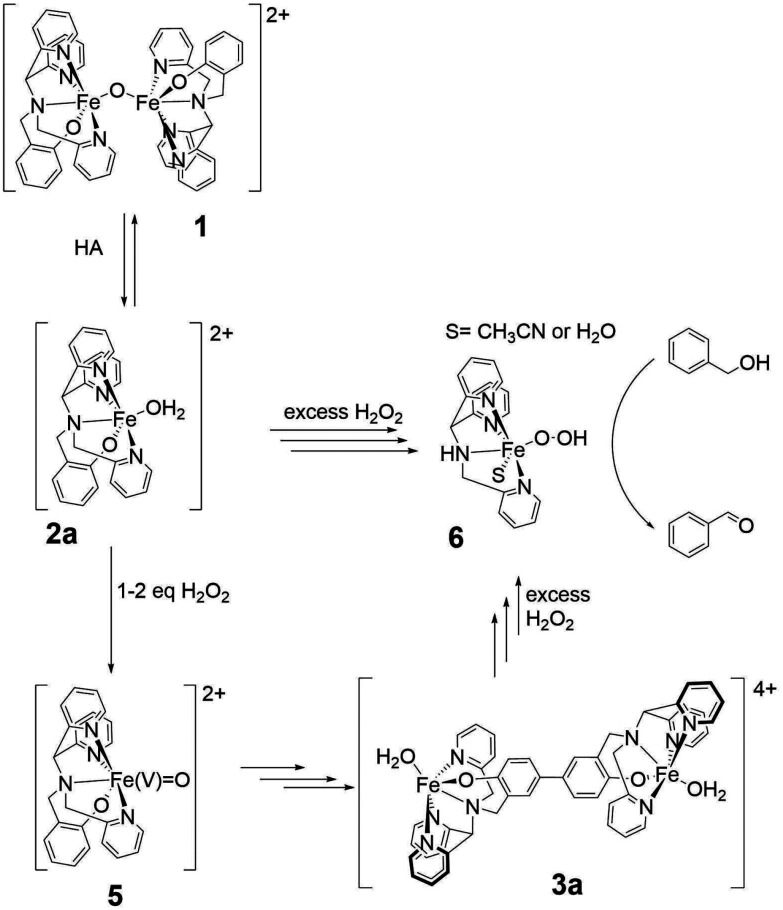
The reaction of 1 with acid (HA) with stoichiometric and excess H_2_O_2_.

Phenolato moieties appear frequently as redox non-innocent moieties in biological systems. For example, in copper dependent enzymes, such as glucose oxidase, they provide the additional redox flexibility required to achieve two-electron oxidation of, *e.g.*, glucose.^50–52^ Similarly, ribonucleotide reductases use a tyrosine residue to provide a stable tyrosyl radical through oxidation by an O_2_ activated non-haem diiron site.^53^ Phenolato moieties have been used in biomimetic iron complexes.^30,54,55^ As a recent example, Goldberg and coworkers^54^ have reported a mononuclear N_5_-Fe(iii)(phenolato)(OH) complex, the reaction of which with the triphenylmethyl radical was used in the study of the mechanism by which Fe(iv)=O species oxidise alkanes (Fig. 2).^56^

**Fig. 3:**
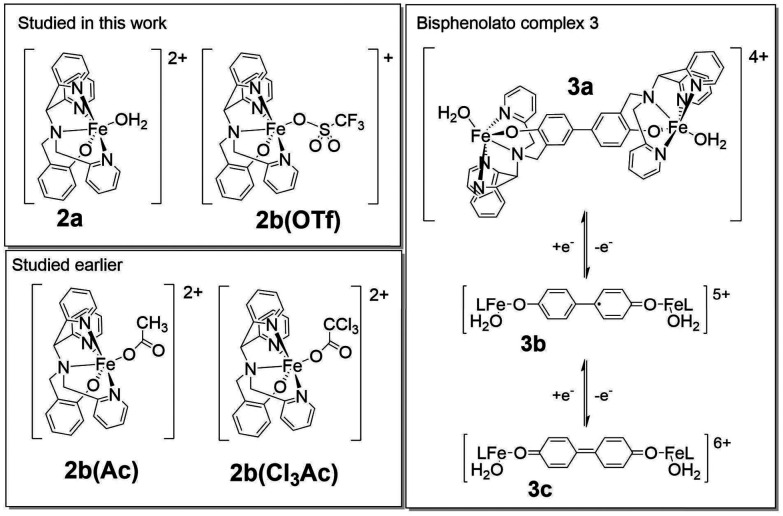
Structures of mononuclear Fe(iii) complexes discussed in the text and redox chemistry of the biphenolato bridged 3.

The complex [(L)Fe(μ-O)Fe(L)]^2+^ (1), where L is 2-(((di(pyridin-2-yl)methyl)(pyridin-2-ylmethyl)amino)methyl)phenol (Fig. 3), contains a phenolato motif and was originally described by Ligtenbarg *et al.* as a catalyst for alcohol oxidation, inspired by the phenolate containing oxidases.^57^ A key observation made by Ligtenbarg *et al.* was that there was a delay between addition of H_2_O_2_ and the start of oxidation of alcohols catalysed by 1, which was removed by the addition of CF_3_SO_3_H.^57^ Recently, we confirmed that the addition of CF_3_SO_3_H removes the delay by breaking up the dinuclear complex 1 to mononuclear complexes (Fig. 2). (*e.g.*, 2a and 2b(OTf) (Fig. 3)). However, we also demonstrated that under catalytic conditions, *i.e.* with excess H_2_O_2_, the phenolato moiety on the ligand detaches rapidly and the oxidation catalysis observed was not due to 1 or 2, but rather due to the complex [(L_1_)Fe(iii)(OH)]^2+^ (6), where L1 is 1,1-di(pyridin-2-yl)-*N*-(pyridin-2-ylmethyl)methanamine (Fig. 2).^58^ The loss of the phenol unit of a related N_3_O phenolato containing iron(iii) complex upon reaction with H_2_O_2_ was suggested earlier also by Ito *et al.*^59^

Although ligand degradation is driven by reaction with excess H_2_O_2_, the initial step is most certainly the reaction of 2 with H_2_O_2_ (Fig. 2). The phenolato moiety of the ligand may be expected to influence O–O bond cleavage in the Fe(iii)–OOH species formed from 2 and H_2_O_2_ and it is of note that the ligand provides an N_4_O coordination environment similar to that provided by the ligands reported by the McKenzie group (*vide supra*).^30^ The increase in electron density on the Fe(iii) centre relative to N_5_ ligands, such as N4Py (Fig. 1) could stabilise high valent products formed upon O–O bond cleavage. Furthermore, Ito *et al.* proposed that interactions between the phenolato π electron cloud and the Fe(iii) coordinated hydroperoxide (*i.e.* the distal oxygen of the peroxide interacts with the π cloud of the aryl ring) could facilitate O–O bond heterolysis.^59^

The accessible redox chemistry of the phenolato moiety is readily apparent in the cyclic voltammetry of 1. The complex shows one electron oxidation, at *ca.* 0.8 V *vs.* SCE, which is a phenoxylato centred oxidation.^60^ Indeed the spin density on the *para*-position of the phenolato ring of the one electron oxidised 1, is sufficient to lead to radical–radical coupling to form C–C bonds between phenolato moieties (*i.e.*, 3a, Fig. 2).^60^ The redox non-innocence that the phenolato moiety of the ligand provides could therefore drive heterolysis of the O–O bond of the Fe(iii)–OOH species to yield a formal Fe(v)=O species (5). The phenoxyl radical character of the ligand could stabilise the Fe(v)=O. Hence, although rapid loss of the phenol unit from the ligand is observed under catalytic conditions with excess H_2_O_2_, these considerations warrant investigation of the first steps in the reaction of 1/CF_3_SO_3_H with near stoichiometric H_2_O_2_.

Here we combine in line reaction monitoring, using simultaneous time resolved UV/vis absorption and resonance Raman spectroscopy, with DFT methods to elucidate the nature of the initial species formed upon reaction of 1 with stoichiometric amounts of H_2_O_2_ in the presence of CF_3_SO_3_H. We show by DFT methods that O–O bond heterolysis in an Fe(iii)(HOOH) species yields initially a formal Fe(v)=O species, better described as a (phenoxyl radical)Fe(iv)=O species, which is facilitated by the stabilization provided by the redox non-innocent phenolato moiety. Experimentally the reaction of H_2_O_2_ with 1 and CF_3_SO_3_H shows initially the product of phenoxyl radical coupling (C–C bond formation at the *para*-position of the phenolates) to generate the same bis-phenol bridged complexes that were observed earlier following electrochemical oxidation of 1.^60^ The initial redox state of this species (*i.e.* doubly oxidised bisphenolato (3c), Fig. 3) provides strong experimental support for the initial formation of a formal Fe(v)=O species.

## Results and discussion

**Fig. 4:**
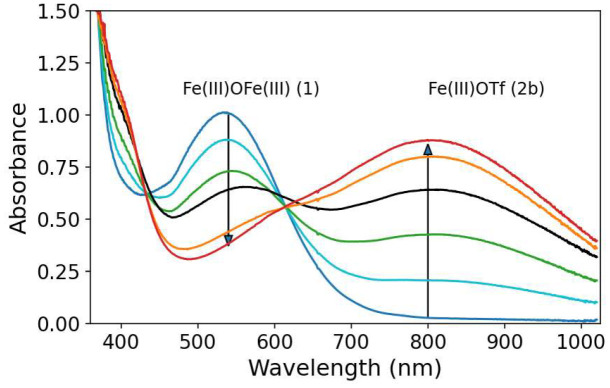
UV/vis absorption spectrum of 1 (0.25 mM) in CH_3_CN (dark blue) and with stepwise addition of 0.3 equiv. CF_3_SO_2_H up to 1.5 equiv. (red).

Addition of 1–2 equiv. CF_3_SO_3_H to 1 yields a mixture of mononuclear complexes (collectively referred to as 2), specifically the aqua (2a) and the triflate (2b) bound complexes (Fig. 3).^58^ In the present study, CF_3_SO_3_H is used since essentially complete conversion of 1 to 2 is observed even when used near-stoichiometrically (Fig. 4).^57,58^

**Fig. 5:**
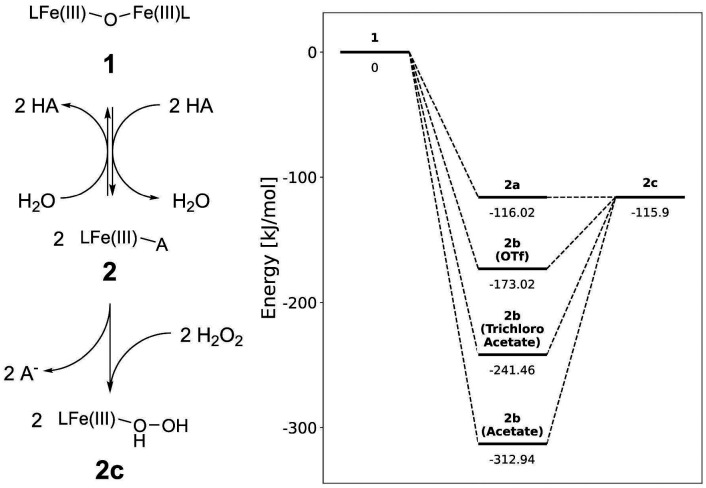
Thermodynamic driving forces for formation of 2 from 1 and subsequent exchange of the conjugate base ligands or H_2_O by H_2_O_2_ in kJ mol^−1^ to form 2c from these species.^58^

In acetonitrile, 1 shows a single band (ligand to metal charge transfer/LMCT) in its UV/vis absorption spectrum with a maximum at 550 nm. The maximum shifts to 800 nm upon addition of CF_3_SO_3_H due to formation of 2 (Fig. 4). An isosbestic point is not maintained consistent with the formation of more than one mononuclear complex.^58^ Subsequent exchange of either H_2_O or CF_3_SO_3_^−^ ligands with H_2_O_2_ is calculated to be only slightly endergonic (*vide infra*) and is found experimentally to be rapid in contrast to other weaker acids are used (Fig. 5 and Table S1).^61^

### UV/vis absorption spectroscopy

**Fig. 6:**
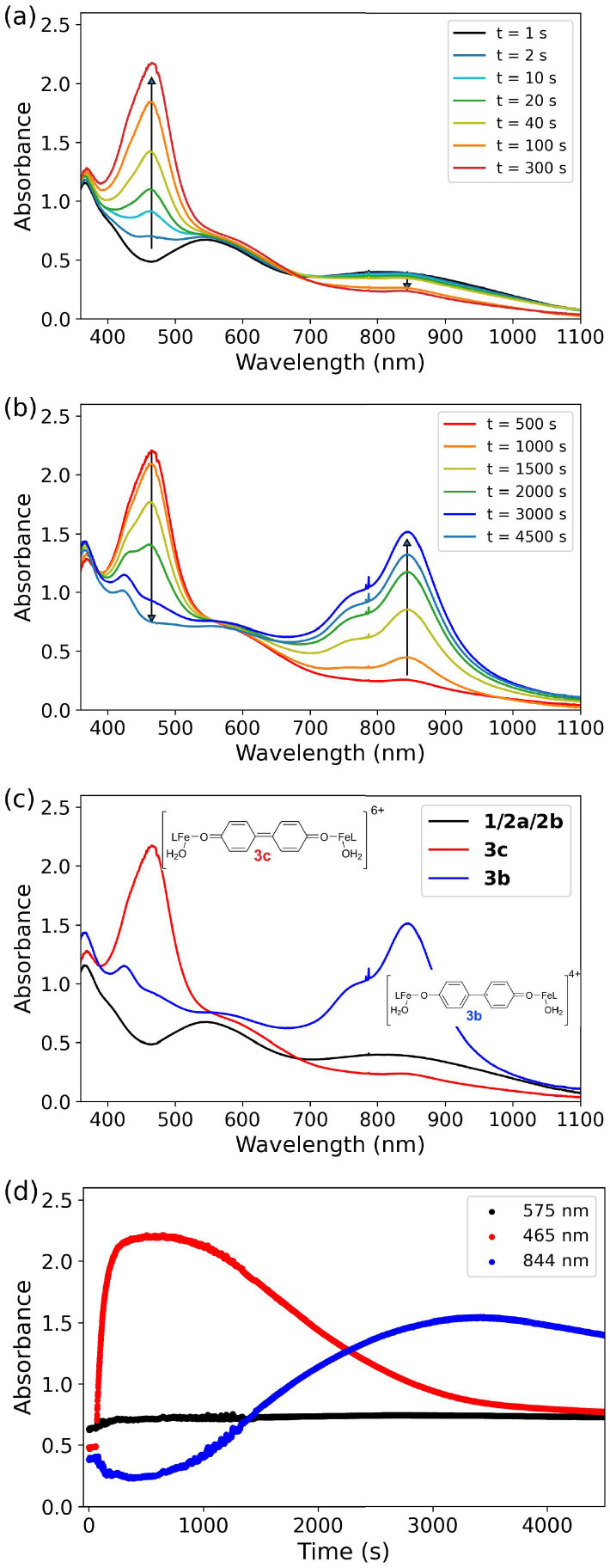
UV/vis absorption spectrum of 1 (0.25 mM) (a) with CF_3_SO_3_H (0.75 equiv., black) and over the first 5 min following addition of H_2_O_2_ (1 equiv.), and (b) between 8.5 and 75 min after. (c) Initial spectrum (black) before addition of H_2_O_2_, and spectra at times where a maximum absorbance at 465 nm (red) and 844 nm (blue) is reached. (d) Absorbance at selected wavelengths over time.

The extent and time dependence of changes observed in the UV/vis absorption spectrum of 1 upon addition of near stoichiometric H_2_O_2_, are highly sensitive to the exact conditions, *i.e.* the number of equiv. CF_3_SO_3_H and H_2_O_2_ added (Fig. S1–S10). Nevertheless, the changes are reproducible and all show the same general trends, exemplified by Fig. 6 for the reaction of 1 and one equiv. CF_3_SO_2_H with one equiv. H_2_O_2_.

Addition of near stoichiometric H_2_O_2_ to 1 with CF_3_SO_3_H results in spectroscopic changes with three distinct stages in all cases. Addition of 1–3 equiv. H_2_O_2_ (50 wt% in water), results in an slight but immediate blue-shift in the absorption maximum due to a decrease in acidity by the water added concomitantly (Fig. 6). This shift is more pronounced when excess water is added with the H_2_O_2_ (*e.g.*, Fig. S1). Thereafter, changes due to reaction of 2 with H_2_O_2_ are observed. In all cases, initially (Fig. 6a) the NIR absorbance decreases slightly and an absorption band at 465 nm appears. The absorbance at *ca.* 465 nm later decreases over time concomitant with appearance of a new band at 844 nm (Fig. 6b). The spectra where maximum absorbance is reached at 465 and 844 nm are shown in Fig. 6c. The 844 nm band persists for some time thereafter (Fig. 6d), and eventually decreases with the spectrum returning to that expected for 1 with acid. The maximum absorbance reached and the duration over which each band is observed varies significantly depending on the precise conditions, however in all cases the same order in appearance and disappearance is observed.^62^ The appearance and decay of the bands at 465 and 844 nm indicate that they are due to distinct species with one species converting into the other (Fig. 6c and d, *vide infra*).

Subsequent addition of water to the reaction mixture results in a decrease in acidity and recovery of a spectrum similar to the initial spectrum of 1, indicating that the phenolato moiety is still bound to the Fe(iii) centre. This recovery in absorption spectrum confirms that the changes observed are not due to the loss of the phenolato moiety from the ligand that occurs when a large excess (>100 equiv. H_2_O_2_) is added.^58^ Furthermore, subsequent additions of H_2_O_2_ lead to further cycles of the same series of changes to the spectra (Fig. S3).

**Fig. 7:**
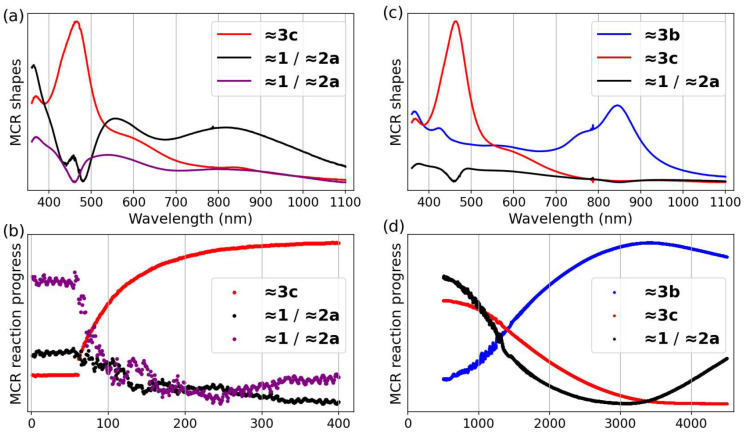
MCR analysis of UV/vis absorption spectra over time following addition of H_2_O_2_ (1 equiv.) to 1 (0.25 mM) with CF_3_SO_3_H (0.75 equiv.). MCR components and their contribution over time are shown (a/b) 0 to 400 s and (c/d) 500 to 4200 s.

Overall the data indicate that addition of H_2_O_2_ results in the formation of four distinct species (1, 3c, 3b and 3a, Fig. 2, *vide infra*) from 2, however, the overlap of the absorption spectra of the various species involved means that the change in absorbance at individual wavelengths (Fig. 6d) does not reflect reaction progress precisely. Therefore, multivariate curve resolution (MCR) analysis was applied to the spectra obtained following addition of H_2_O_2_ to 1/CF_3_SO_3_H (Fig. 7).

Analysis of the first period of the reaction (*i.e.* until a maximum absorbance at 465 nm is reached) is complicated by the initial slight shift in the absorption spectrum of 2 due to concomitant addition of water with the H_2_O_2_ (Fig. 7a and b). Nevertheless, the data in this period can be explained by three components, two of which correspond broadly to a combination of the spectra of 1 and 2, and a third component that resembles closely the expected spectrum of 3c (Fig. 7a), *vide infra*. In the second period, as the absorbance at 465 nm decreases again, the data can be explained well by three components resembling 1/2, 3c, and 3b (Fig. 7c and d).

### Resonance Raman spectroscopy

**Fig. 8:**
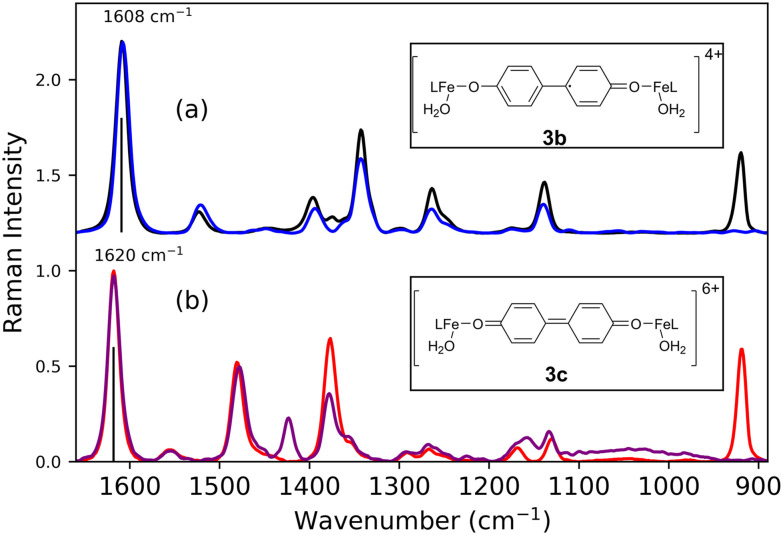
Raman spectra at (a) *λ*_ex_ 785 nm (black, 165 s, Fig. 9) and (b) *λ*_ex_ 473 nm (red, 2380 s, Fig. 10) of 1 with CF_3_SO_3_H (0.75 equiv.) after addition of H_2_O_2_ (1 equiv.) in CH_3_CN, with spectra obtained upon electrochemical oxidation of poly-1 at (a) *λ*_ex_ 785 nm (blue) and (b) *λ*_ex_ 488 nm (purple).^60^

The bands observed in the absorption spectra during the reaction correspond closely to those observed earlier during spectroelectrochemical studies of poly-1.^60^ In dichloromethane, electrochemical oxidation of 1 results in C–C coupling of the phenoxylate moieties, which together with the Fe–O–Fe moiety result in formation of a polymer on electrode surfaces (poly-1). This polymer can undergo electrochemical oxidation to poly-1^1+^ and subsequently to poly-1^2+^. Each state shows well defined UV/vis-NIR absorption (Fig. S11) and resonance Raman spectra (Fig. 8), which allow for assignment of the species observed here (*vide supra*). Specifically the NIR absorption band at 844 nm is observed also for poly-1^+^ and the absorption band at 465 nm in the spectrum of poly-1^2+^ (see Fig. S11 for comparison of UV/vis absorption spectra).

Raman spectra recorded at *λ*_exc_ 473 nm and *λ*_exc_ 785 nm at the moment of maximum absorption (at 465 nm and 844 nm, respectively) correspond exactly to the resonance Raman spectra (Fig. 8) of the electrochemically generated poly-1^2+^ and poly-1^+^, respectively, reported earlier,^60^ providing definite assignment of the spectra to these 4,4′-bisphenolato species (Fig. 3).^63^ The presence of CF_3_SO_3_H means that the Fe–O–Fe bridge in poly-1^60^ is not present in the complexes observed here. However, since the electronic transitions and therefore the absorption and resonance Raman spectra, are localised to the 4,4′-biphenolato moiety in its doubly and singly oxidised states, Raman bands associated with other ligand components on the iron centre are not observed. Furthermore, although the Raman spectra obtained at *λ*_exc_ 473 and 785 nm are broadly similar, they show distinct differences in band positions and relative intensity. The bands at 1608 and 1620 cm^−1^ can be used as marker bands^60^ for one- and two-electron oxidised bis-phenolato moiety of 3b and 3c, respectively.

### Reaction progress monitoring with resonance Raman spectroscopy

**Fig. 9:**
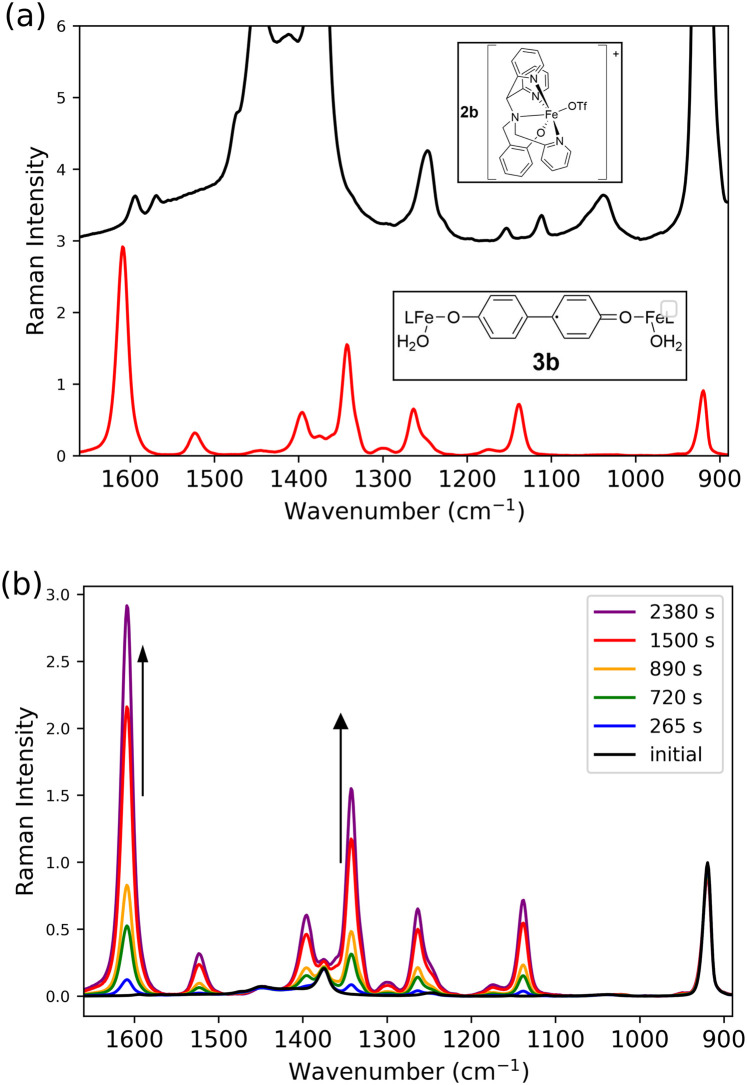
Raman spectrum at *λ*_exc_ 785 nm (a) before (black) and 2380 s (red) after addition of H_2_O_2_ (1 equiv.) to 1 (0.25 mM) with CF_3_SO_3_H (0.75 equiv.). Spectra are vertically offset and rescaled for clarity (see Fig. 6 for UV/vis absorption spectra). (b) From 0 (black) to 2380 s. Spectra were normalised with respect to the intensity of the Raman band of CH_3_CN at 920 cm^−1^, and baseline corrected.

**Fig. 10:**
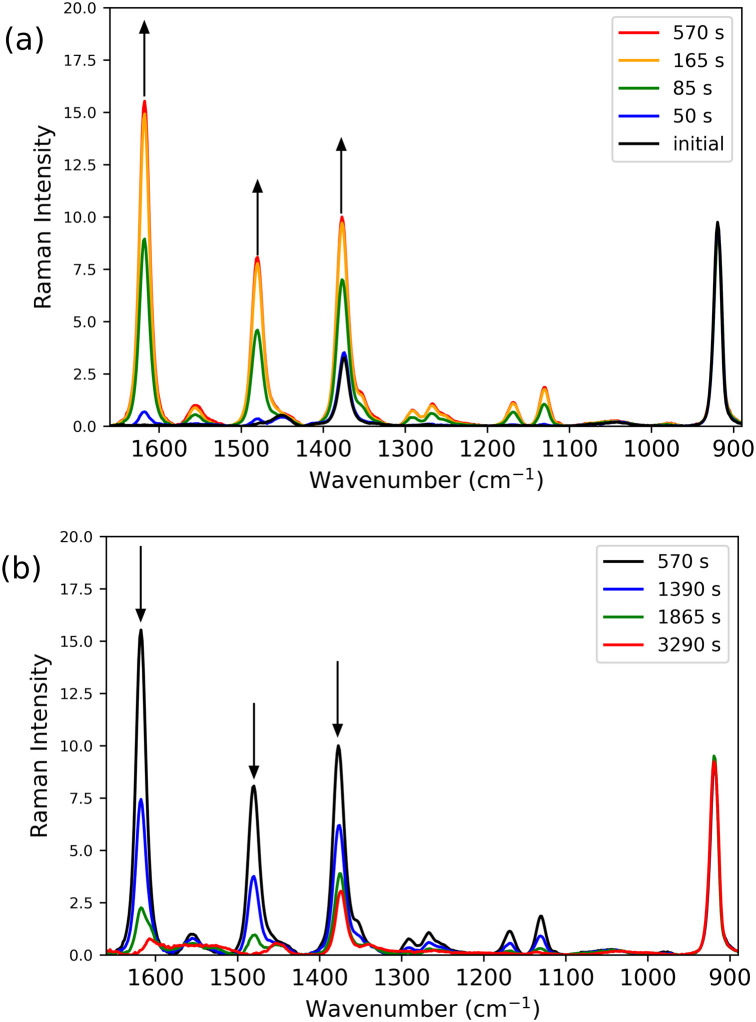
Raman spectrum at *λ*_exc_ 473 nm over time following addition of H_2_O_2_ (1 equiv.) to 1 (0.25 mM) with CF_3_SO_3_H (0.75 equiv.) (see Fig. 6 for UV/vis absorption spectra) (a) from 0 (black) to 570 s and (b) from 570 to 3290 s. Note that although the spectrum is dominated by Raman scattering from 3c (*e.g.*, band at 1620 cm^−1^), the characteristic band of 3b at 1608 cm^−1^ is observed in the final spectrum (red). Spectra were normalised with respect to the intensity of the Raman band of CH_3_CN at 920 cm^−1^, and baseline corrected.

Although MCR analysis indicates that initially only 3c is formed and that 3b is generated upon subsequent one electron reduction, the overlapping and shifting of absorption bands creates an uncertainty in the time dependence of the appearance and disappearance of the species involved. Since many of the Raman bands of the species involved are well resolved, Raman spectroscopy at 473 nm and 785 nm allow for the relative concentrations of the various species to be followed over time. Reaction progress monitored by Raman spectroscopy at 473 nm allows for tracking of 3c (*λ*_max_ 465 nm), whereas at 785 nm the disappearance of 2b (OTf) (with a broad band around 800 nm) and appearance of 3b (*λ*_max_ 844 nm) can be followed. Since the time dependence of changes in UV/vis absorption spectra were found to be quite sensitive to the exact amount of CF_3_SO_3_H and H_2_O_2_ added, Raman spectra at both wavelengths of excitation (785 and 473 nm, Fig. 9 and 10, respectively) were recorded simultaneously with UV/vis absorption spectra (Fig. 6).

**Fig. 11:**
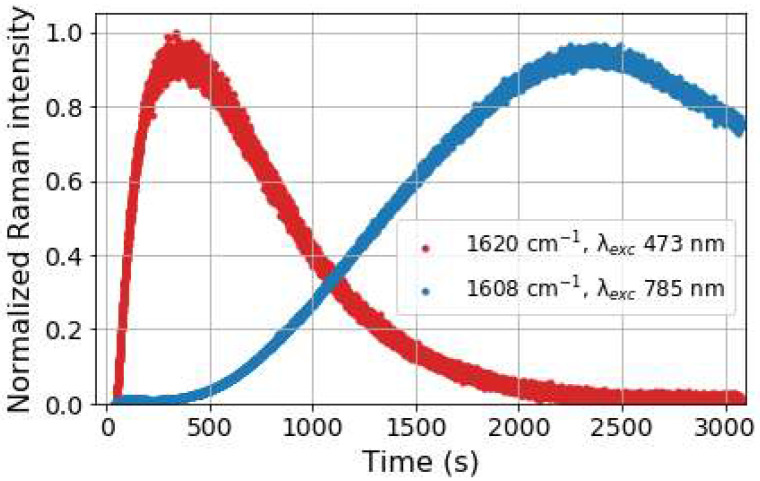
Raman intensity over time following addition of H_2_O_2_ (1 equiv.) to 1 (0.25 mM) with 0.75 equiv. CF_3_SO_3_H in CH_3_CN at 1620 cm^−1^ (blue, *λ*_exc_ 473 nm) and 1608 cm^−1^ (red *λ*_exc_ 785 nm). Intensities are corrected for inner filter effects.

**Fig. 12:**
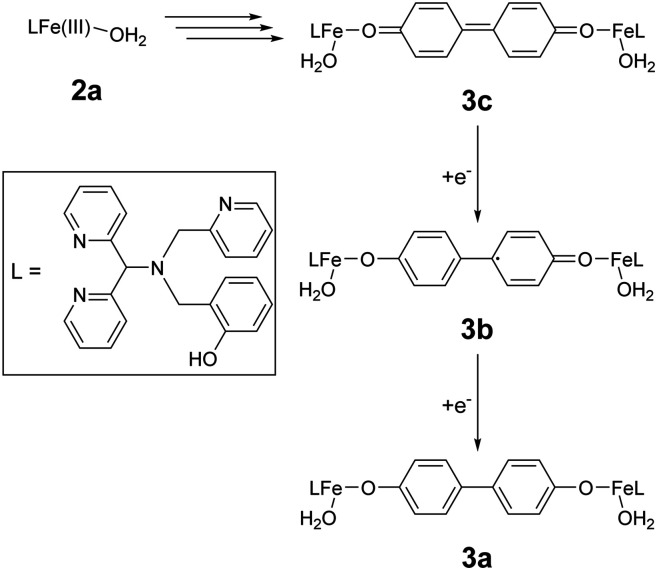
The experimentally established species present during the reaction of 1 and CF_3_SO_3_H with H_2_O_2_.

Raman spectra recorded at 785 nm, show initially the rapid disappearance of the resonantly enhanced Raman bands of 2b (OTf) (Fig. 9a, black), with a subsequent slow increase in intensity at 1608 cm^−1^ (*i.e.*3b) over 500 s after which the intensity increased rapidly over the following 2000 s and thereafter decrease slowly (Fig. 9b and 11). In contrast, the Raman spectrum at 473 nm (Fig. 10a) shows only Raman scattering from the solvent before H_2_O_2_ was added where upon the intensity of the Raman bands (*e.g.*, 1620 cm^−1^) of 3c increases immediately and reached a maximum within 400 s. Thereafter the intensity of this band decreases over time (Fig. 10b). Towards the end of the reaction the marker band of 3b at 1608 cm^−1^ becomes apparent first as a shoulder on the 1620 cm^−1^ band and eventually only the 1608 cm^−1^ band is observed (Fig. 10b and S12) consistent with the increase and decrease in the Raman scattering of 3b observed at *λ*_exc_ 785 nm. The intensity of the Raman bands at 1620 and 1608 cm^−1^ (at *λ*_exc_ 473 nm and 785 nm, respectively) increase and decrease (Fig. 11) concomitant with absorbance at 465 and 844 nm, respectively (Fig. 7). The Raman spectral data confirm that 3c is formed initially and its later reduction leads to the appearance of 3b (Fig. 10b and 12).

In summary, the data show a rapid initial build up in concentration of 3c is followed by subsequent reduction to 3b and eventually to species resembling 1 and 2 (Fig. 12). The formation of these species, and more specifically the order of their formation, with first 3c and only later 3b, raises a question as to the mechanism by which they are formed during the reaction with H_2_O_2_. Possible reaction pathways leading from 2 to 3b and 3c were explored computationally.

### Thermodynamic analysis of reaction pathways

Computational (DFT) methods were used to estimate the driving forces for the individual steps in the reaction of H_2_O_2_ with 1 in the presence of a Brønsted acid. The driving force for dissociation of 1 into mononuclear complexes (2) with various acids was described earlier.^58^ Experimentally, addition of CF_3_SO_3_H to 1 results in protonation and spontaneous dissociation of the Fe(iii)–O–Fe(iii) bridge to form mononuclear complexes, *e.g.*, 2a and 2b(OTf) (Fig. 3).

Note that we have studied all three possible spin states (low, intermediate and high spin) for each of the species, and report here the energies corresponding to the lowest spin states of the species. The multiplicities of the species are listed in the SI.

Addition of H_2_O_2_ to mixtures of 2a/2b (OTf) results in exchange with the H_2_O and CF_3_SO_3_^−^ ligands (Fig. 5, left) to yield 2c. The exchange is endergonic (Fig. 5, right), but only modestly so when CF_3_SO_2_H is used, and thus the equilibrium would be expected to be rapid enough to allow the subsequent reactions to proceed at a rate sufficient for intermediates to be observed (*vide infra*). Hence, 2a is taken as a starting point for calculations as it is present regardless of which acid is used.

### Barriers to O–O bond cleavage

**Fig. 13:**
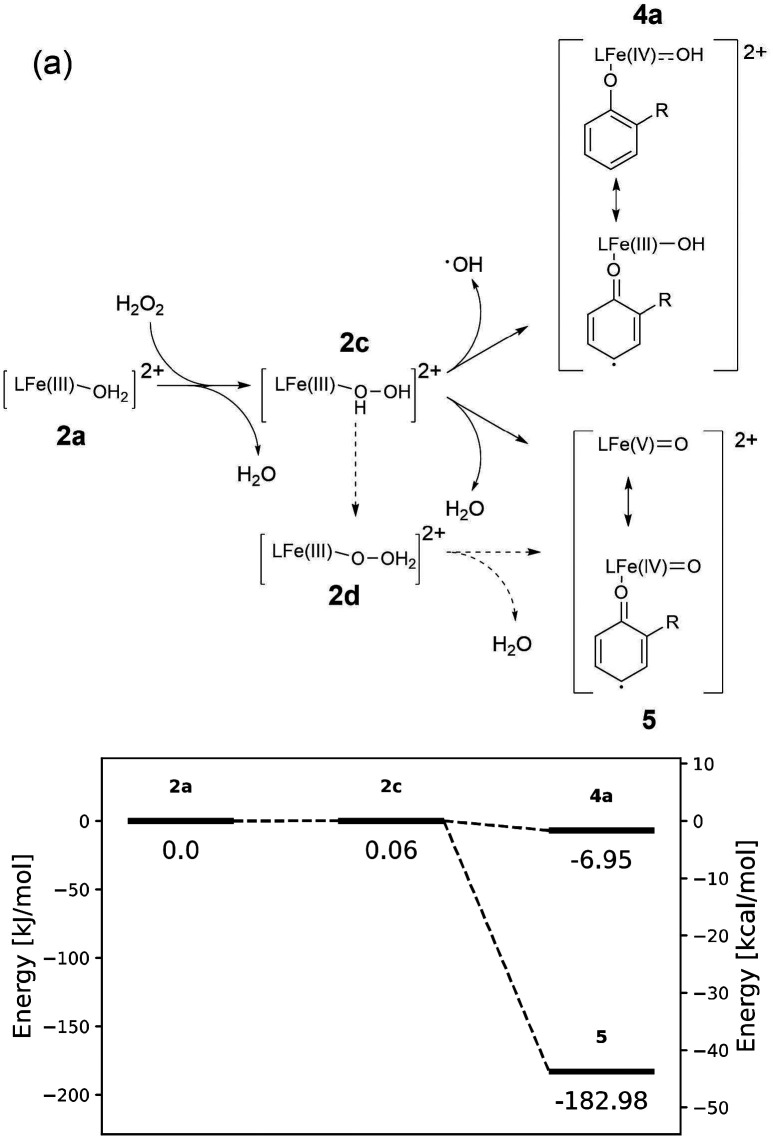
(a) Possible pathways following exchange of H_2_O with H_2_O_2_ and subsequent dissociation of the O–O bond in 2c and 2d. (b) Calculated energies for species formed by homolytic and heterolytic cleavage of the O–O bond of 2c. All energies are relative to the aqua complex (2a).

Three reaction paths that follow from formation of 2c are considered here (Fig. 13). Specifically, homolytic cleavage of the O–O bond of (i) Fe(iii)(H_2_O_2_) (2c) or (ii) Fe(iii)(OOH) (2e), and (iii) heterolytic cleavage of the O–O bond of Fe(iii)(H_2_O_2_) (2c), with pathway (i) and (ii) experimentally most reasonable due to the presence of strong acid. The kinetic competence of the pathway was evaluated based on the energies of the intermediates leading to O–O bond cleavage. Linear transit calculations used to find transition state structures are reported in the SI (Fig. S13).

#### Homolytic dissociation

For the structurally related complex [(N4Py)Fe(iii)OOH]^2+^ (Fig. 1) several energy barriers to homolytic O–O bond cleavage have been reported, each depending on the presence of other molecules in the 2^nd^ coordination sphere.^64^ The Gibbs-free energy barrier to homolysis reported recently by Chen *et al.* is 38.16 kJ mol^−1^,^65^ which decreases to 13.39 kJ mol^−1^ by inclusion of an explicit molecule of H_2_O_2_ in the 2^nd^ coordination sphere (simulating high concentrations of H_2_O_2_) proximal to the hydroperoxido ligand. The inclusion of the additional molecule of H_2_O_2_ leads to overall disproportion to O_2_ and two molecules of H_2_O.^64,66^

In the present study, homolytic dissociation of the O–O bond of 2c in the presence of near stoichiometric amounts of H_2_O_2_ (*i.e.* where it is unlikely that a second molecule of H_2_O_2_ will be in proximity to the Fe(iii)–(H)OOH moiety) would result in formation of a formal Fe(iv)–OH 4a (Fig. 13). The barrier to O–O bond dissociation for 2c is 26.80 kJ mol^−1^, which is substantially lower than the barrier to O–O bond dissociation (39.92 kJ mol^−1^) in 2e (Fe(iii)–OOH) (*vide infra*). However, both barriers are sufficiently low for the reactions to proceed rapidly at room temperature.

#### Heterolytic dissociation

Heterolytic dissociation starting from the distal protonated H_2_O_2_ bound intermediate is barrierless, with spontaneous dissociation to a formal Fe(v)=O species (5) and H_2_O. Starting from the initially formed proximally protonated hydroperoxide intermediate (2c), transfer of the proton proximal to the iron to the distal peroxide oxygen (2d) occurs prior to O–O bond cleavage. This proton transfer, likely mediated by protic species present in solution (water, acid, *etc*.), occurs in combination with the O–O bond dissociation. The reaction is facilitated by the large driving force for the overall process.

### Dissociation pathways

Although the barrier to homolytic cleavage is low enough to proceed rapidly at room temperature, the lack of any barrier to heterolytic O–O bond cleavage together with the large thermodynamic driving force suggests the latter reaction is most likely to dominate at room temperature. We therefore consider all three of the homolytic and heterolytic pathways.

### Homolytic dissociation of the Fe(iii)–HOOH intermediate

**Fig. 14:**
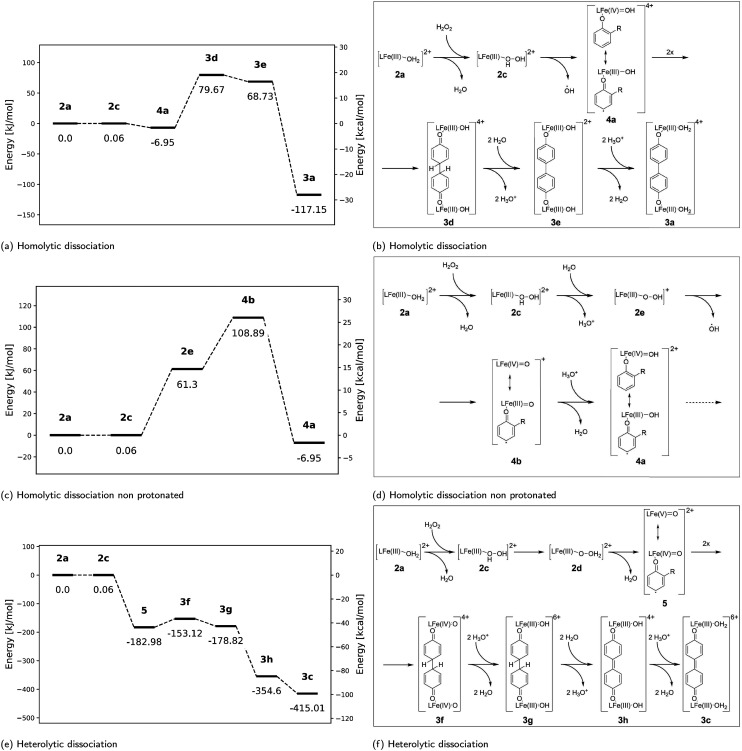
(left) Calculated Gibbs free energies (kJ mol^−1^) for the (a) homolytic, (c) homolytic (non-protonated H_2_O_2_) and (e) heterolytic O–O bond cleavage pathway and subsequent coupling pathways possible thereafter. All energies are relative to 2a. (right) Proposed pathway for (a) homolytic, (c) homolytic (non-protonated H_2_O_2_) and (e) heterolytic O–O bond cleavage and the subsequent coupling pathways possible thereafter. Energies indicated in (a), (c) and (d) are in kJ mol^−1^. The second y-axis indicates energy in kcal mol^−1^ for reference.

Homolytic dissociation of 2c yields formally a protonated Fe(iv)=O species 4a (Fig. 14b). The formation of 4a is slightly favourable, but the subsequent coupling reactions are energetically uphill resulting in a significant barrier to an overall thermodynamically favourable path to 3a (Fig. 14a). The experimentally observed 3c does not arise naturally from this coupling pathway, as it requires subsequent two electron oxidation. This can only be achieved *via* Fe(iv)=O by reaction with H_2_O_2_ twice. This path would require a build-up first of 3b, and only latter of 3c, which is not observed. Hence the pathway is unlikely to be relevant experimentally. Furthermore, analysis of spin density (*vide infra*) indicates that the product of initial C–C coupling is relatively unstable.

### Homolytic dissociation of the Fe(iii)–OOH intermediate

Despite the acidity of the acetonitrile solution in which the reaction takes place, it is worth considering the energy of the non-protonated structures and pathways and to contrast them to the pathways followed by the protonated complexes (Fig. 14b and f). Fig. 14d shows the path that can be followed after deprotonation of 2c to form 2e. Deprotonation of 2c is thermodynamically unfavourable (Fig. 14c). Although subsequent reprotonation of the Fe(iv)=O (4b) is thermodynamically favourable (Fig. 14c), 4b is unlikely to form in the reaction mixture. Nevertheless, it is useful to consider this pathway for comparison with the experimentally relevant complexes 4a and 5 (*vide infra*).

### Heterolytic dissociation of the Fe(iii)–HOOH intermediate

Heterolytic cleavage of the O–O bond in 2d results in the spontaneous formation of a formal Fe(v)=O species (5, Fig. 14f). Although subsequent C–C bond formation is slightly uphill, the formation of 3c that follows is sufficiently favourable to drive the reaction forward (Fig. 14e). The formation of 3c, which contains the doubly oxidised biphenolate moiety, is consistent with the first major species observed experimentally by UV/vis absorption and resonance Raman spectroscopy with near-stoichiometric amounts of H_2_O_2_. Of note also is the transition from 3f to 3g to 3h. Here, protonation to form 3g, and subsequent deprotonation to form 3h is more energetically favourable than *vice versa via* the Fe(iv)=O compound 4b (Fig. S14).

### Spin density and aryl aryl (C–C) coupling

**Fig. 15:**
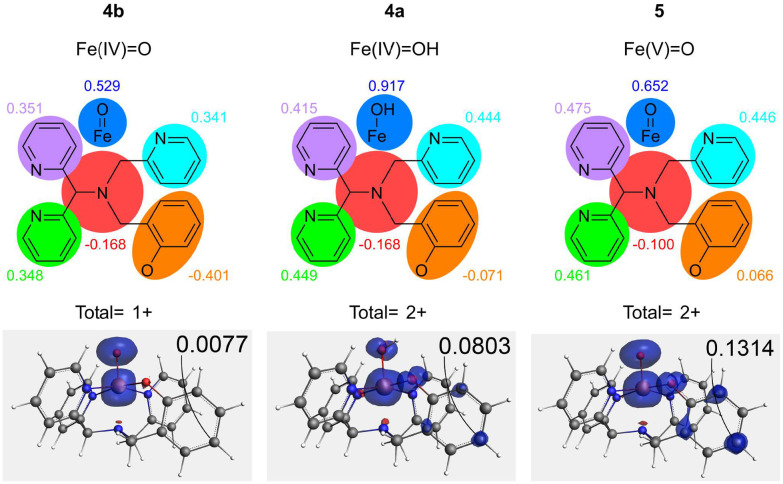
The Mulliken charge densities summed for fragments (top) and spin densities (bottom, shown in blue transparent, isosurface 0.003) of the complexes generated upon O–O bond cleavage in the hydroperoxido intermediates. The MDC-m spin density on the *para* phenolate carbon (where C–C bond formation takes place) is indicated in the top right corner of each structure. The structures are shown schematically, in order from left to right: 4b, 4a and 5.

The charge and spin density distribution over the complexes formed by O–O bond cleavage in the hydroperoxido intermediates was examined to better understand the driving forces for the O–O bond cleavage itself and for the observed subsequent C–C coupling between phenol units (Tables S2–S8). The charge density in 4b (Fig. 15, top left) shows that the ligand backbone donates some electron density to the Fe–O unit. In contrast, the spin density analysis indicates that the spin density is centred on the Fe=O unit, with little spin density spreading over the ligand backbone. This state is similar to that of the complex [(N4Py)Fe(iv)=O]^2+^. The negligible spin density in 4b, except that observed on the Fe=O unit (Fig. 15, bottom left), shows that 4b should have little propensity to engage in radical–radical coupling reactions on the phenolate unit. Protonation of 4b to give 4a shifts the excess spin density significantly to the phenolate unit, enhancing reactivity towards radical reactions such as C–C coupling at the *para* position of the phenolate ring. This spread is not quite as extensive as in 5 (*vide infra*), but is significantly more so than in the non-protonated 4b.

The charge density in 4a shows that the formal Fe(iv)=OH structure is more accurately described as an Fe(iii)–OH species with an oxidized ligand backbone (Fig. 15, top centre). Furthermore, analysis of the charge density in the ligand backbone shows that, as for 5, the phenolato moiety is relatively neutral and the N3Py unit donates the majority of the charge to the iron centre. The spin density in 4a, which is spread throughout some of the ligand backbone, is especially centred in the phenolate moiety on the oxygen and the carbon atoms *ortho* and *para* to the phenolate oxygen (Fig. 15, bottom centre).

For 5, charge density analysis shows that the formal Fe(v) structure is more accurately described as an Fe(iv)=O (Fig. 15, top right). Separate analysis of the charge density in the ligand backbone shows that the phenolato moiety is relatively neutral, and that the remaining N3Py unit donates the majority of the charge to the iron centre.

The spin density in 5 is spread out partially over the ligand backbone, especially the phenolato oxygen and the carbons *ortho* and *para* to the oxygen of the phenolato moiety, and hence it is better described as a phenoxyl radical (Fig. 15, bottom right). Unlike 4b, the excess spin in 5 spreads significantly to the phenolate unit, enhancing reactivity towards radical reactions such as phenolate-phenolate coupling at the *para* position of the phenolato moiety.

### Summary

The heterolytic O–O bond cleavage pathway is, computationally, the most favourable of the possible cleavage pathways, and the cleavage product 5 has ample spin density on the *para* position of the phenolato ring to facilitate rapid C–C bond formation between two molecules of 5. The biphenolato moieties formed following double deprotonation are more easily oxidised than the original phenolato moiety,^60^ which triggers concomitant internal electron transfer to generate an Fe(iii)–OR centre with the biphenol bridge being in the two-electron oxidized state. It is this species (3c) that is observed first experimentally. Later reduction leads to the one-electron oxidised species (3b) and then the fully reduced Fe(iii) bridged complexes that have similar spectroscopic properties to 1/2. The role of the phenolato in driving heterolytic bond cleavage is therefore in large part due to the stabilisation of the formal Fe(v)=O species formed upon heterolytic O–O bond cleavage.

## Conclusions

The observation of 3c, and subsequent observation of 3b, after addition of 1 equiv. H_2_O_2_ to 1, with near stoichiometric triflic acid, provides strong evidence towards a heterolytic O–O bond cleavage pathway that yields a formal Fe(v)=O species. In contrast to the O–O bond homolysis seen in N5–Fe complexes, such as [(N4Py)Fe(iii)(OOH)]^2+^, the presence of the redox non-innocent phenolato ligand facilitates heterolysis to a formal Fe(v)=O state by spreading spin and charge density over the ligand components.

The induction of peroxy O–O bond heterolysis by the redox non-innocence of the phenolato ligand gives promise to phenolato-based iron complexes similar to 1 as oxidation catalysts, provided that competing degradation *via* loss of the phenolato moiety can be avoided.^58^ The use of redox active ligands therefore can be seen as an opportune approach to the efficient use of H_2_O_2_ in organic oxidations catalysed by non-haem iron complexes. Importantly, this approach avoids the formation of ROS such as the hydroxyl radical and thereby increase selectivity. Future studies will focus on overcoming the ligand degradation, that occurs when excess (>10 equiv.) H_2_O_2_ is used, by adapting the ligand or conditions.

## Experimental

### Materials

Solvents and reagents were obtained from commercial suppliers and used as received. 50% w/w H_2_O_2_ in water was obtained from Aldrich. The complex 1 was reported earlier and the detailed synthesis and characterisation of the complex is described by Unjaroen *et al.*^58,60^ Reaction of 1 with H_2_O_2_ was typically carried out by addition of 5–10 μL of stock solutions of H_2_O_2_ (50% v/v) and of CF_3_SO_3_H in CH_3_CN to 1 (0.28 mM in CH_3_CN) in quartz cuvettes.

### Instrumentation

UV/vis absorption spectra were recorded in 1 cm path length quartz cuvettes using a Specord600 (AnalytikJena) or an AvaSpec-ULS2048CL-EVO spectrometer fibre coupled to a cuvette holder and a halogen light source (HL-2000-FHSA). Raman spectra at 785 nm were recorded using a 785 nm fibre coupled laser (400 mW at sample, Cobolt 08-NLDM) with a Raman probe (Avantes) fibre coupled to a Shamrock 163i spectrograph and idus-420-OE CCD with a 600 l mm^−1^ grating blazed at 700 nm. Raman spectra at 473 nm were recorded in 180° backscattering mode. Excitation was provided by a free space 50 mW 473 nm laser (Cobolt lasers), which was guided to the optical path of the spectrometer by a dichroic mirror and focused on the sample with a 5 cm focal length planoconvex lens. Backscattered light was collected through the same objective and passed through the dichroic beam splitter and a Rayliegh line rejection filter before being focused into a Shamrock 303i spectrograph with a 1200 l mm^−1^ grating blazed at 500 nm and a Newton EMCC-470DU CCD detector (Andor Technology) operating in CCD mode. Spectra were acquired using Andor SOLIS, with spectral calibration using cyclohexane (ASTM E1840, DOI: 10.1520 E1840-96R22) and processed using Spectragryph and Python. Primary inner filter effects due to the large changes in absorbance at the wavelengths of excitation were corrected for by normalisation of the Raman spectra at 800 cm^−1^ (Raman band of the solvent CH_3_CN). Correction for secondary inner filter effects is complicated by differences in absorbance across the wavelength range of the Raman spectrum that are proportional over time. Therefore Raman spectra at 473 nm and 785 nm were recorded concurrently with UV/vis absorption spectroscopy (*vide supra*), to allow for correction for the secondary inner filter effect (reabsorption of Raman scattering).

### DFT methods

As described earlier,^58,60^ the Amsterdam Density Functional (ADF), using the Amsterdam Modelling Suite (AMS) (version 2022.1)^67,68^ and QUILD routines, were used in the optimization of structures with S12g/TZ2P,^69^ including solvation and relativistic corrections, which include the Grimme D3^70^ dispersion model. Molecular orbitals were expanded in an uncontracted set of Slater type orbitals (STOs) of triple-ζ quality with double polarization functions (TZ2P).^71^ Core electrons are not treated explicitly during the geometry optimizations (frozen core approximation). Molecular density was fit using an auxiliary set of s, p, d, f, and g STOs to represent the Coulomb and exchange potentials accurately for each SCF cycle. Geometries of all possible spin states were optimized with the AMS driver using adapted delocalized coordinates until the maximum gradient component was less than 10^–4^ a.u. in all cases. Energies, gradients and Hessians (for vibrational frequencies) were calculated using S12g,^69^ by including solvation effects through the COSMO dielectric continuum model with appropriate parameters for acetonitrile.^72–74^ Note that both dispersion, solvation and relativistic effects were included self-consistently in all calculations.^75^ Scalar relativistic corrections were included self-consistently using the zeroth-order regular approximation (ZORA). For all calculations carried out with S12g the Becke grid of VeryGood quality was used, except for analytical frequencies which used the Normal grid. All DFT calculations were performed using the unrestricted Kohn–Sham scheme, and in the case of anti-ferromagnetically coupled dinuclear systems, the ModifyStartPotential approach within ADF was used. Several possible spin states were considered for each complex.

## Author contributions

WRB, DU and DRD concieved the project. DRD, DU, CMdR, WRB and MDBM acquired experimental data. DRD and MS acquired DFT data. All authors contributed to the analysis of the data and writing of the manuscript.

## Conflicts of interest

There are no conflicts to declare.

## Supplementary Material

DT-054-D5DT01477H-s001

## Data Availability

Data is provided as SI. Original data available from corresponding authors. Supplementary information: Additional spectroscopic data and coordinates for all calculated structures. See DOI: https://doi.org/10.1039/d5dt01477h.
