# Supplementary material for: Ligand driven heterolytic O–O bond cleavage in a non-haem phenolato-Fe(iii)–OOH complex to yield a formal Fe(v)[double bond, length as m-dash]O intermediate
Source: Dalton Trans. 2025 Sep 8;54(38):14566–77. doi: 10.1039/d5dt01477h (PMC12435456; doi:10.1039/d5dt01477h)
Supplement: DT-054-D5DT01477H-s001 [file DT-054-D5DT01477H-s001.pdf]

Supplementary Information (SI) for Dalton Transactions.
This journal is © The Royal Society of Chemistry 2025

Electronic Supplementary Information
Ligand Driven Heterolytic O-O Bond Cleavage in a non-haem Phenolato-Fe(III)-OOH Complex to Yield a Formal Fe(V)=O intermediate

Daniël R. Duijnstee,[a,b] Marika Di Berto Mancini,[a] C. Maurits de Roo,[a] Duenpen Unjaroen,[a] Moniek Tromp,[b] Ronald Hage,[a] Wesley R. Browne,*[a] and Marcel Swart,*[c,d]

[a] *Stratingh Institute for Chemistry, Faculty of Science and Engineering, University of Groningen, Nijenborgh 3, 9747AG, Groningen, The Netherlands.*

[b] *Zernike Institute for Advanced Materials, Faculty of Science and Engineering, University of Groningen, Nijenborgh 3, 9747AG, Groningen, The Netherlands.*

[c] *IQCC & Dept. Química, Universitat de Girona, Campus Montilivi, 17003 Girona, Spain.*

[d] *ICREA, Pg. Lluís Companys 23, 08010 Barcelona, Spain.* *corresponding authors. Wesley R. Browne, Marcel Swart
email: w.r.browne@rug.nl, marcel.swart@udg.edu

Driving force for ligand exchange with H_2O_2

Table S1 Thermodynamic driving forces for formation of 2 from 1 and subsequent exchange of the conjugate base ligands or H_2O by H_2O_2 in kJ/mol. Driving forces for formation of 2 from other species reported earlier.[58] The driving force for exchange of H_2O_2 is discussed in further detail below.

Acid/Ligand at exchangeable site	ΔG for **2** formation	ΔG for H_2O_2 exchange
$CH_3CO_2H/CH_3CO_2^-$	-312.94	98.52
$CCl_3CO_2H/CCl_3CO_2^-$	-241.46	62.78
$CF_3SO_3H/CF_3SO_3^-$	-173.02	28.56
H_2O	-116.02	0.06

Reaction of 1 and CF_3SO_3H or acetic acid with H_2O_2

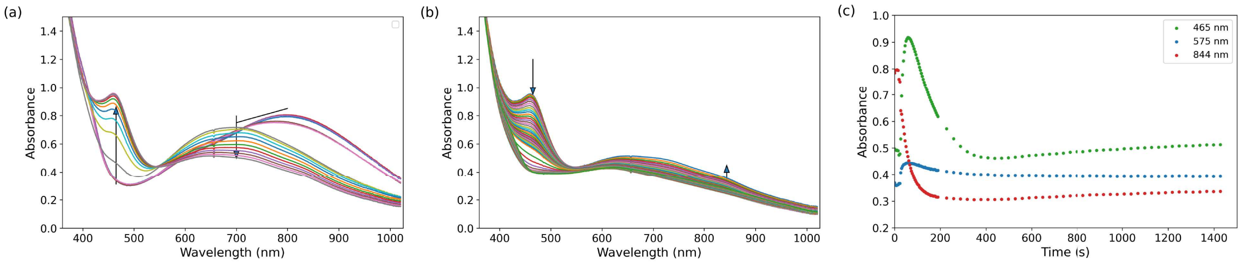

Figure S1 UV/vis absorption spectra over time (a) 0 to 75 and (b) 75 to 1470 s following addition of H_2O_2 (one equiv.) to 1 with CF_3SO_3H (one equiv). (c) Absorbance at 465 nm (green), 575 nm (blue) and 844 nm (red) over time. Note that the H_2O_2 was diluted 1000 fold in water before addition.

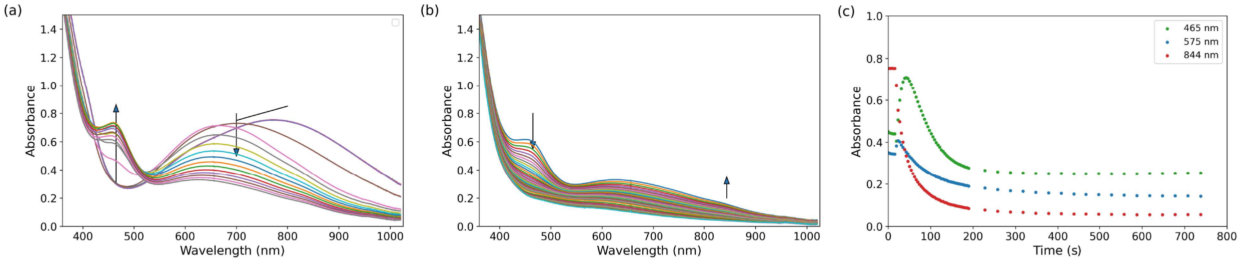

Figure S2 UV/vis absorption spectra over time (a) 0 to 75 and (b) 75 to 1470 s following addition of H_2O_2 (two equiv.) to 1 with CF_3SO_3H (one equiv). (c) Absorbance at 465 nm (green), 575 nm (blue) and 844 nm (red) over time. Note that the H_2O_2 was diluted 1000 fold in water before addition.

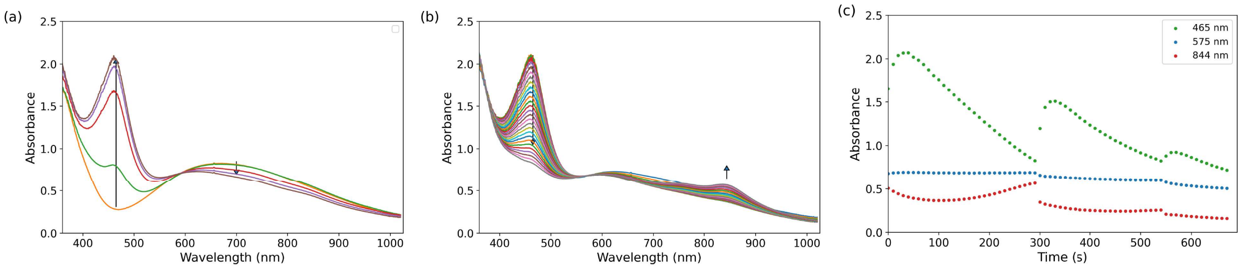

Figure S3 UV/vis absorption spectra over time (a) 0 to 40 and (b) 40 to 300 s following addition of H_2O_2 (two equiv.) to 1 with CF_3SO_3H (one equiv) and 5 μL H_2O . (c) Absorbance at 465 nm (green), 575 nm (blue) and 844 nm (red) over time, showing changes following three sequential additions of H_2O_2 (2 equiv. each).

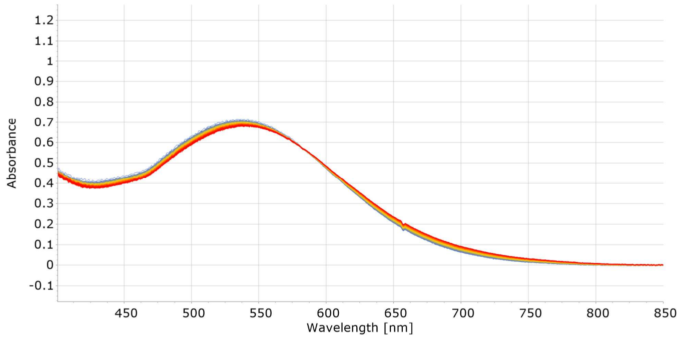

Figure S4 1 (0.25 mM) in CH_3CN with 70 equiv. acetic acid (blue), before and after addition of 2 eq of H_2O_2 (red, after ca. 25 min)

Addition of H_2O_2 dilute in water to 1

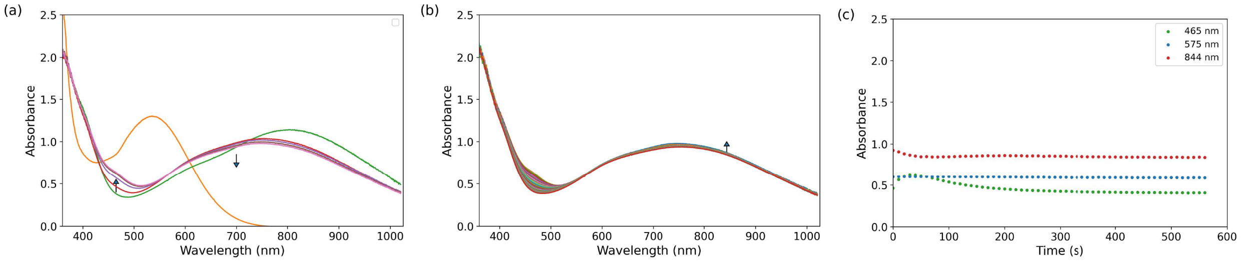

Figure S5 UV/vis absorption spectra over time (a) 0 to 40 and (b) 40 to 300 s following addition of H_2O_2 (0.5 equiv.) to 1 with CF_3SO_3H (one equiv). (c) Absorbance at 465 nm (green), 575 nm (blue) and 844 nm (red) over time. Note that H_2O_2 was diluted 1000 fold in water before addition.

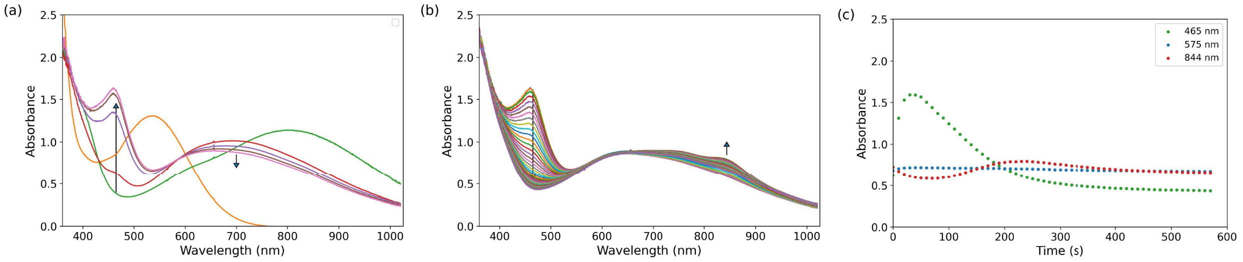

Figure S6 UV/vis absorption spectra over time (a) 0 to 40 and (b) 40 to 300 s following addition of H_2O_2 (one equiv.) to 1 with CF_3SO_3H (one equiv). (c) Absorbance at 465 nm (green), 575 nm (blue) and 844 nm (red) over time. Note that H_2O_2 was diluted 1000 fold in water before addition.

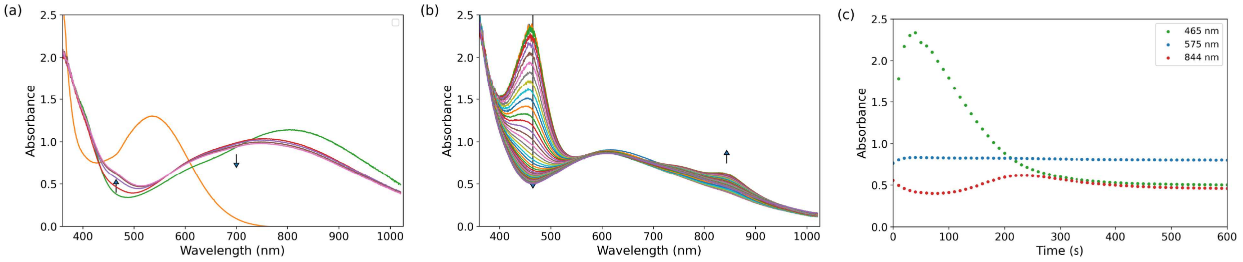

Figure S7 UV/vis absorption spectra over time (a) 0 to 40 and (b) 40 to 300 s following addition of H_2O_2 (1.5 equiv.) to 1 with CF_3SO_3H (one equiv). (c) Absorbance at 465 nm (green), 575 nm (blue) and 844 nm (red) over time. Note that H_2O_2 was diluted 1000 fold in water before addition.

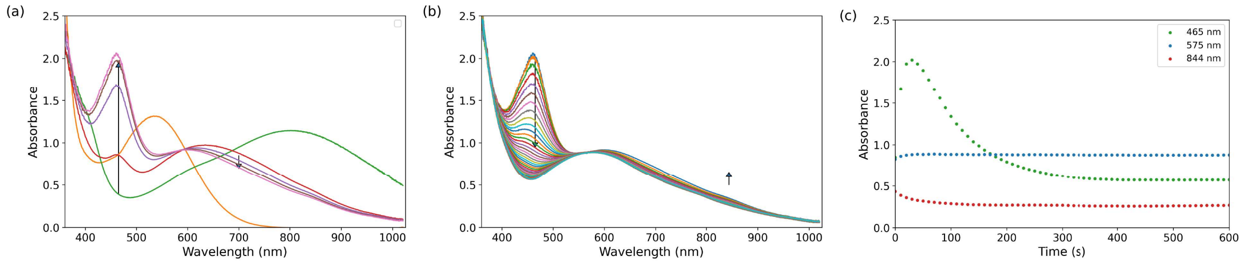

Figure S8 UV/vis absorption spectra over time (a) 0 to 40 and (b) 40 to 300 s following addition of H_2O_2 (two equiv.) to 1 with CF_3SO_3H (one equiv). (c) Absorbance at 465 nm (green), 575 nm (blue) and 844 nm (red) over time. Note that H_2O_2 was diluted 1000 fold in water before addition.

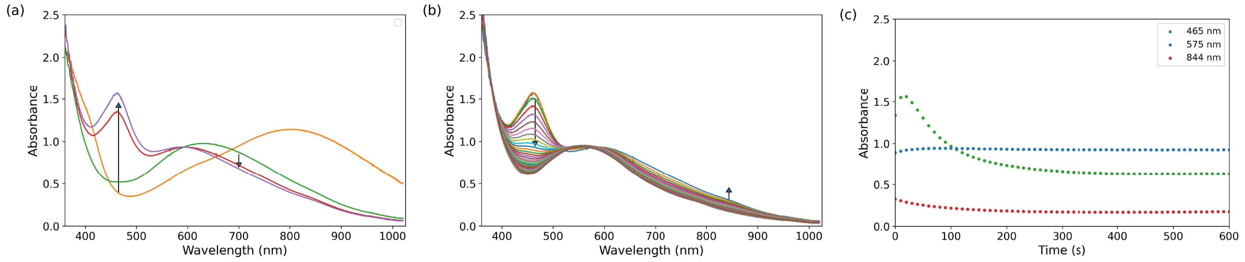

Figure S9 UV/vis absorption spectra over time (a) 0 to 40 and (b) 40 to 300 s following addition of H_2O_2 (two equiv.) to 1 with CF_3SO_3H (one equiv). (c) Absorbance at 465 nm (green), 575 nm (blue) and 844 nm (red) over time. Note that H_2O_2 was diluted 1000 fold in water before addition.

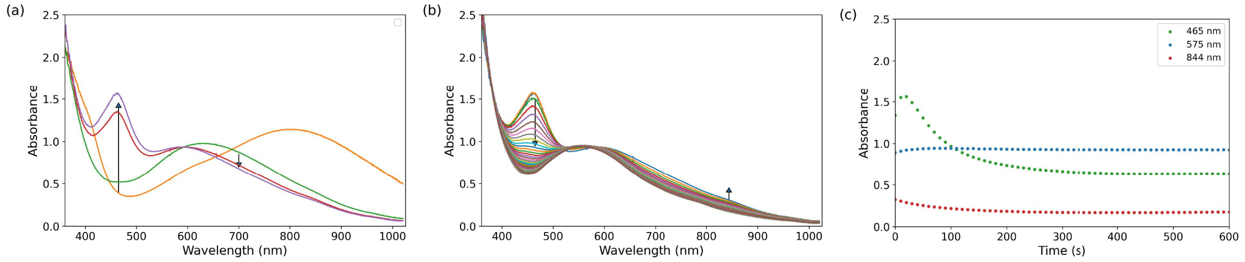

Figure S10 UV/vis absorption spectra over time (a) 0 to 40 and (b) 40 to 300 s following addition of H_2O_2 (three equiv.) to 1 with CF_3SO_3H (one equiv). (c) Absorbance at 465 nm (green), 575 nm (blue) and 844 nm (red) over time. Note that H_2O_2 was diluted 1000 fold in water before addition.

Raman spectra of 1/CF_3SO_3H with H_2O_2 at λ_{exc} 473 and 785 nm

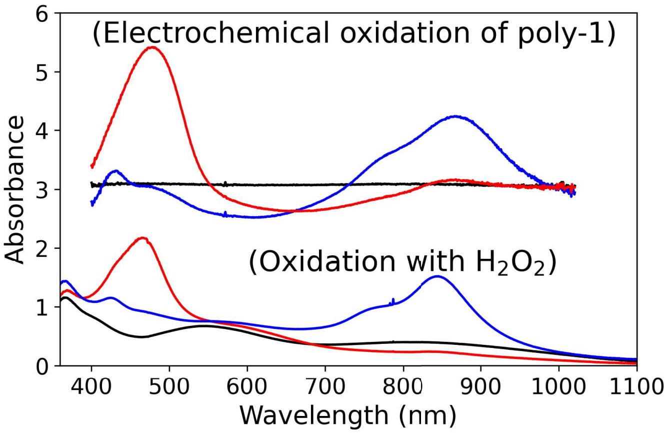

Figure S11 UV/vis absorption spectra of 1 with CF_3SO_3H (0.75 equiv., black) and at times where a maximum absorbance at 465 nm (red) and 844 nm (blue) is reached following addition of H_2O_2 (1 equiv.). Difference spectra obtained during electrochemical oxidation of thin films of poly-1 on ITO on glass at 0.0, 0.65 and 0.8.0 V are shown offset and scaled by 50 times for comparison. See reference[60] for details.

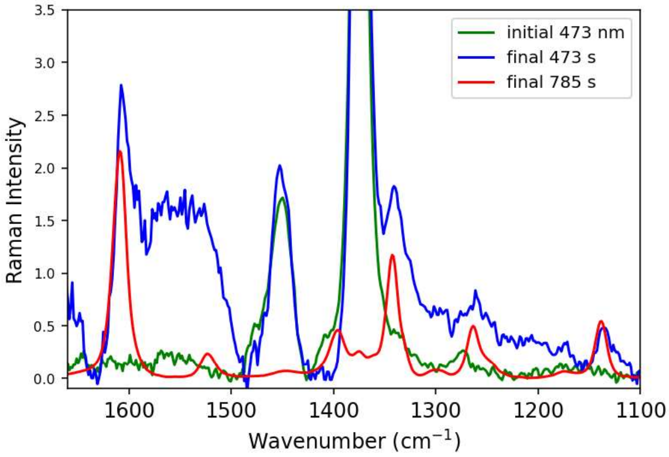

Figure S12 Raman spectra at (green) before and (blue) 4000 s after addition of 1 eq H_2O_2 to 1 with CF_3SO_3H at λ_{exc} 473 nm (see figure 10) and 4000 s after at at λ_{exc} 785 nm (see figure 9).

Linear transits to barriers for dissociation

The dissociation barrier values shown in Figure S13 are higher than reported in the main text since a vibrational correction to the energy is not applied. Vibrational corrections were only carried out for the transition state structures and thus is not represented specifically in these plots.

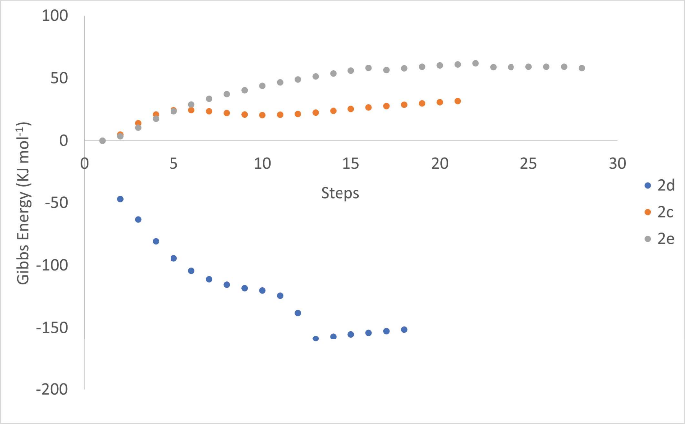

Figure S13 Energies per step during linear transits for O-O bond cleavage in 2c, 2d and 2e

Order of protonation and deprotonation steps in going from 3f to 3h

The step between **3f** and **3h** could involve protonation first via **3g** or deprotonation first via **3i**, followed by deprotonation or protonation respectively. The protonation first via **3g** is the most favourable, not introducing an energy barrier to the reaction unlike reaction via **3i**.

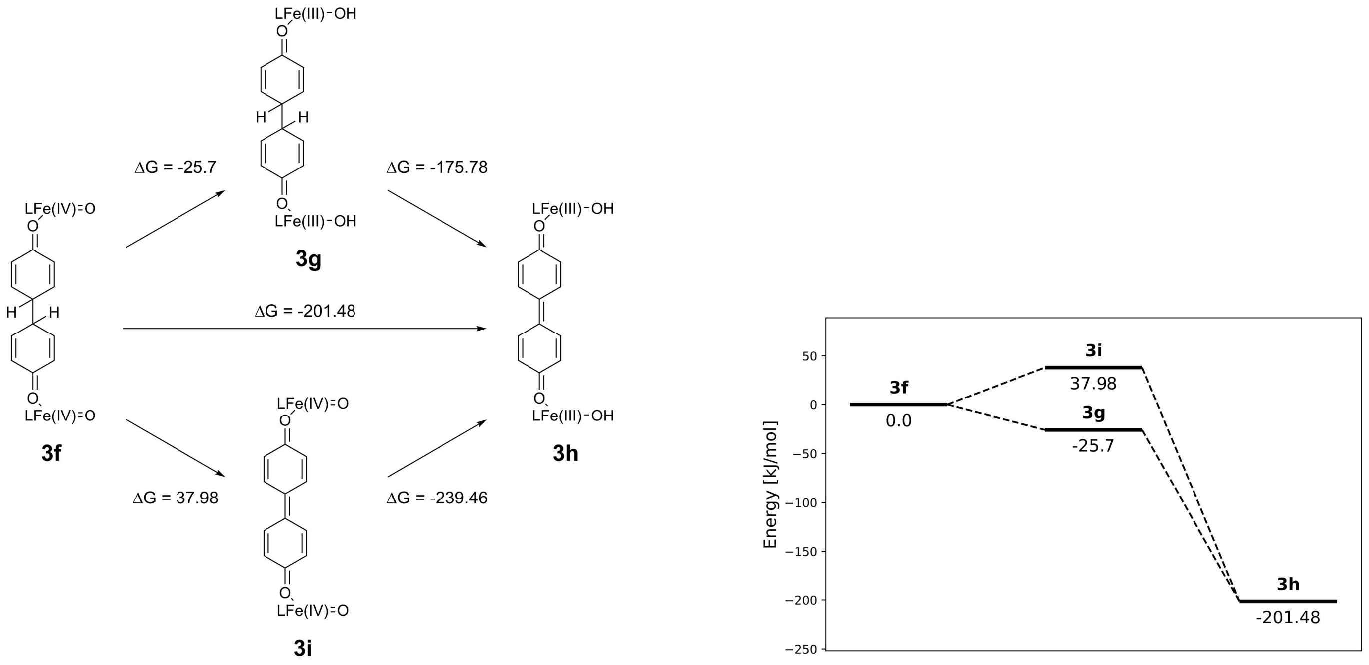

Figure S14 Left: Possible pathways following C-C coupling of 5 to form 3f. Right: Calculated energies for species formed by protonation and subsequent deprotonation via 3g, and vice versa via 3i.

Spin and charge density
Spin density

Table S2 The MDC-m spin densities of parts of the complexes generated from dissociation of the hydroperoxido intermediates.

Atom	Fe(IV)=O	Fe(V)=O	Fe(IV)=OH
total	2	3	2
Fe-oxo unit (full)	2.0716	2.4608	1.6542
Fe	1.4007	1.7046	1.4478
O (oxo)	0.6709	0.7561	0.1996
O (phenolate)	-0.0014	0.2187	0.1715
C (ortho, inner)	-0.0060	0.0749	0.0495
C (ortho, outer)	-0.0068	0.0888	0.0593
C (para)	-0.0077	0.1314	0.0803
Ligand (full)	-0.0716	0.5392	0.3458
Phenolato	-0.0186	0.5440	0.3730
Non-Phenolato	-0.0531	-0.0047	-0.0272

Fe(IV)=O

Table S3 The Mulliken charge densities of parts of the Fe(IV)-oxo complex generated from homolytic dissociation of the hydroperoxido intermediate

Structure	Charge (Mulliken)
Total	0.9998
Fe-O	0.5175
Ligand backbone	0.4823
Phenolate backbone	-0.4130
Non-phenolate backbone	0.8953

Table S4 The MDC-m spin densities of parts of the Fe(IV)-oxo complex generated from homolytic dissociation of the hydroperoxido intermediate

Atom	Spin density (MDC-m)
Fe	1.400715
O (oxo)	0.670927
O (phenolate)	-0.001449
C (ortho, backbone)	-0.006016
C (ortho, outer)	-0.006834
C (para)	-0.007723
Fe-oxo unit	2.071642
Ligand backbone	-0.071641
Phenolate backbone	-0.018555
Non-Phenolate backbone	-0.053086

Fe(V)=O

Table S5 The Mulliken charge densities of parts of the Fe(V)-oxo complex generated from heterolytic dissociation of the protonated hydroperoxido intermediate

Structure	Charge (Mulliken)
Total	1.9995
Fe-O	0.6521
Ligand backbone	1.3474
Phenolate backbone	0.0656
Non-phenolate backbone	1.2818

Table S6 The MDC-m spin densities of parts of the Fe(V)-oxo complex generated from heterolytic dissociation of the protonated hydroperoxido intermediate

Atom	Spin density (MDC-m)
Fe	1.704634
O (oxo)	0.756141
O (phenolate)	0.218725
C (ortho, backbone)	0.074902
C (ortho, outer)	0.088802
C (para)	0.131405
Fe-oxo unit	2.460775
Ligand (full)	0.539226
Phenolato	0.543951
Non-Phenolato	-0.004725

Table S7 The Mulliken charge densities of parts of the protonated Fe(IV)-oxo complex generated from homolytic dissociation of the protonated hydroperoxido intermediate

Structure	Charge (Mulliken)
Total	2.0006
Fe-O	0.9165
Ligand backbone	1.0841
Phenolate backbone	-0.0705
Non-phenolate backbone	1.1546

Table S8 The MDC-m spin densities of parts of the protonated Fe(IV)-oxo complex generated from homolytic dissociation of the protonated hydroperoxido intermediate

Atom	Spin density (MDC-m)
Fe	1.447759
O (oxo)	0.199597
O (phenolate)	0.171497
C (ortho, backbone)	0.049514
C (ortho, outer)	0.059292
C (para)	0.080324
Fe-oxo unit	1.654165
Ligand backbone	0.345834
Phenolate backbone	0.373006
Non-Phenolate backbone	-0.027172

Fe(IV)=OH

Bond length rationalization

The bond lengths of the phenolate C-O and phenolate-phenolate C-C bonds can be compared to reference compounds (Table S9) to rationalize why the Fe(IV)=O dimer is higher in energy than the protonated phenolate structure that comes before it (Figure 14f and 14e). The trend in bond lengths indicates that the protonated phenolate structures have more C=O double bond character, which serves to rationalize their relative stability.

Table S9 Bond lengths of representative bonds of the protonated phenolato moieties, their corresponding deprotonated structures, and reference compounds.

	diphenol	(prot) benzoquinone	difference
O(phen)-C	1.363	1.240	-0.123
C-C(center)	1.474	1.595	0.121
	oxo	prot_oxo	difference
O(phen)-C	1.324	1.26	-0.064
C-C(center)	1.469	1.596	0.127
	hydroxy	prot_hydroxy	difference
O(phen)-C	1.322	1.261	-0.061
C-C(center)	1.467	1.617	0.15

O-O dissociation pathway energies

Homolytic cleavage

Table S10 The individual thermodynamic driving forces for the steps in the homolytic dissociation pathway

Homolytic dissociation	kJ/mol	kcal/mol
1	0.06	0.014
2	-7.01	-1.68
3	86.62	20.70
4	-10.94	-2.61

Heterolytic cleavage

Table S11 The individual thermodynamic driving forces for the steps in the heterolytic dissociation pathway

Heterolytic dissociation	kJ/mol	kcal/mol
1	0.06	0.01434036
2+3	-183.04	-43.74765824
4	29.86	7.13671916
5	37.98	9.07744788
6	-239.46	-57.23237676

Homolytic cleavage of non-protonated peroxide

Table S12 The individual thermodynamic driving forces for the steps in the homolytic dissociation pathway of the non-protonated hydroperoxido intermediate

non-prot hom diss	kJ/mol
1	0.06
2	61.24
3	47.59
4	-115.84

Calculations used for computational thermodynamic analysis of reaction pathways

Method

The equation for each reaction, with Gibbs free energies obtained from frequency calculations.
For each reaction:

$$n \cdot A + m \cdot B + \cdots \rightarrow x \cdot C + y \cdot D + \cdots \tag{4}$$

A Gibbs free energy equation is set up:

$$\Delta G = x \cdot C + y \cdot D + \cdots - (n \cdot A + m \cdot B + \cdots) \tag{5}$$

In the following section, only the reaction equation is shown for the sake of brevity.

Ligand exchange with H_2O_2

2f → **2c**:

$$\mathbf{2f} + H_2O_2 \rightarrow \mathbf{2c} + CH_3CO_2{}^- \tag{6}$$

2g → **2c**:

$$\mathbf{2g} + H_2O_2 \rightarrow \mathbf{2c} + CCl_3CO_2{}^- \tag{7}$$

2b → **2c**:

$$\mathbf{2g} + H_2O_2 \rightarrow \mathbf{2c} + CF_3SO_3{}^- \tag{8}$$

2a → **2c**

$$\mathbf{2a} + H_2O_2 \rightarrow \mathbf{2c} + H_2O \tag{9}$$

Homolytic dissociation of the protonated hydroperoxido intermediate

2a → **2c**

$$\mathbf{2a} + H_2O_2 \rightarrow \mathbf{2c} + H_2O \tag{10}$$

2c → **4a**

$$\mathbf{2c} \rightarrow \mathbf{4a} + \cdot OH \tag{11}$$

4a → **3d**

$$2 \cdot \mathbf{4a} \rightarrow \mathbf{3d} \tag{12}$$

3d → **3e**

$$\mathbf{3d} + 2 \cdot H_2O \rightarrow \mathbf{3e} + 2 \cdot H_3O^+ \tag{13}$$

3e → **3a**

$$\mathbf{3e} + 2 \cdot H_3O^+ \rightarrow \mathbf{3a} + 2 \cdot H_2O \tag{14}$$

Homolytic dissociation of the non-protonated hydroperoxido intermediate

2a → **2c**

$$\mathbf{2a} + H_2O_2 \rightarrow \mathbf{2c} + H_2O \tag{15}$$

2c → **2e**

$$\mathbf{2c} + H_2O \rightarrow \mathbf{2e} + H_3O^+ \tag{16}$$

2e → **4b**

$$\mathbf{2e} \rightarrow \mathbf{4b} + \cdot OH \tag{17}$$

Table 1 Lowest energy spin state complexes. (OS = open shell)

**1**	OS $S = 0$						
**2a**	$S = 5/2$	**2b**	$S = 5/2$	**2f**	$S = 5/2$	**2g**	$S = 5/2$
**2c**	OS $S = 1/2$	**2d**	$S = 1/2$	**2e**	$S = 1/2$		
**3a**	OS $S = 0$	**3b**	OS $S = 0$	**3c**	OS $S = 0$		
**3d**	OS $S = 0$	**3e**	OS $S = 0$				
**3f**	OS $S = 0$	**3g**	OS $S = 0$	**3h**	OS $S = 0$		
**4a**	$S = 1$	**4b**	$S = 1$	**5**	$S = 3/2$		

4b → **4a**

$$\mathbf{4b} + H_3O^+ \rightarrow \mathbf{4a} + H_2O \tag{18}$$

Heterolytic dissociation of the protonated hydroperoxido intermediate

2a → **2c**

$$\mathbf{2a} + H_2O_2 \rightarrow \mathbf{2c} + H_2O \tag{19}$$

2c → **5**

2d spontaneously decomposes to **5** due to the combination of a large driving force and a simple reaction path of elongation of the O-O bond. The comparison is started with the stable structure of **2c**, to obtain an energy for the reaction, and from this the overall driving force of rearrangement of the proton and the dissociation of the O-O bond is calculated.

$$\mathbf{2c} \rightarrow \mathbf{5} + H_2O \tag{20}$$

5 → **3f**

$$2 \cdot \mathbf{5} \rightarrow \mathbf{3f} \tag{21}$$

3f → **3h**

$$\mathbf{3f} \rightarrow \mathbf{3h} \tag{22}$$

3h → **3c**

$$\mathbf{3h} + 2 \cdot H_3O^+ \rightarrow \mathbf{3c} + 2 \cdot H_2O \tag{23}$$

Lowest energy spin states

Coordinates

All coordinates are provided in .xyz format

1

103
Open-shell singlet (S = 0)
Fe 1.695100 -0.416100 0.131500
Fe -1.689700 0.422100 0.185300
H -5.664000 0.640300 -0.663100
C -4.441000 1.146100 1.465100
H -3.812500 1.673200 2.190600
H -5.428200 1.630400 1.466800
C -4.567400 -0.290600 1.866100
C -5.815300 -0.859600 2.108200
H -6.706500 -0.245500 1.981400
C -5.943100 -2.188800 2.501800
H -6.927600 -2.614900 2.681300
C -4.795200 -2.965500 2.658600
H -4.879400 -4.007900 2.961900
C -3.541300 -2.420600 2.423300

H -2.641700 -3.024000 2.530700
C -3.398100 -1.080000 2.018100
O -0.002400 0.003700 -0.177400
O 2.238400 0.583300 1.708100
N 3.810400 -1.299900 0.010200
N 1.521000 -2.368600 1.058300
N 1.784900 -1.802100 -1.715300
N 2.781900 1.008700 -1.073900
C 0.542600 -2.751300 1.885000
H -0.240000 -2.018200 2.077100
C 0.518000 -4.021700 2.444500
H -0.294300 -4.306800 3.107300
C 1.546200 -4.906900 2.141800
H 1.558700 -5.906200 2.569600
C 2.561700 -4.504000 1.279900
H 3.378700 -5.170600 1.017200
C 2.509600 -3.223300 0.753100
C 3.480600 -2.705300 -0.290900
H 4.368400 -3.350500 -0.337100
C 2.716800 -2.757000 -1.598900
C 2.899700 -3.740600 -2.560100
H 3.670400 -4.496300 -2.433700
C 2.074900 -3.728600 -3.680600
H 2.190600 -4.484400 -4.453400
C 1.104300 -2.740500 -3.799400
H 0.439000 -2.699800 -4.657200
C 0.997100 -1.792100 -2.790800
H 0.250600 -1.002100 -2.827900
C 2.279500 2.226500 -1.317800
H 1.227800 2.361500 -1.072800
C 3.046500 3.256200 -1.833100
H 2.599300 4.228500 -2.019800
C 4.394400 3.013500 -2.085500
H 5.033400 3.801200 -2.477300
C 4.917500 1.757300 -1.818000
H 5.968800 1.539700 -1.988500
C 4.079700 0.767700 -1.312700
C 4.570500 -0.631600 -1.058500
H 4.458800 -1.196500 -1.990300
H 5.643400 -0.619900 -0.832800
C 4.482800 -1.150200 1.324800
H 3.877100 -1.688500 2.061400
H 5.470700 -1.631900 1.290900
C 4.617900 0.281300 1.741100
C 5.871000 0.849500 1.956800
H 6.759600 0.239000 1.797500
C 6.007600 2.173600 2.364100
H 6.995900 2.599200 2.522200
C 4.863200 2.945800 2.562900
H 4.954000 3.984400 2.877400
C 3.604200 2.401600 2.354800
H 2.707200 3.001700 2.494800
C 3.451900 1.066100 1.936100
O -2.190300 -0.596100 1.763300
N -3.807000 1.311000 0.133600
N -1.486900 2.364300 1.128800

N -1.828700 1.829800 -1.642700
N -2.812000 -0.984600 -1.008400
C -0.484500 2.736800 1.930900
H 0.301900 2.000300 2.092300
C -0.441600 4.001200 2.503000
H 0.389400 4.278200 3.145700
C -1.475800 4.891400 2.238500
H -1.473900 5.886300 2.676800
C -2.516400 4.499200 1.401700
H -3.338900 5.170200 1.169000
C -2.482100 3.223900 0.860800
C -3.482900 2.719100 -0.160900
H -4.370400 3.366300 -0.175100
C -2.756100 2.784000 -1.489600
C -2.965500 3.777600 -2.434900
H -3.731600 4.532500 -2.278600
C -2.173300 3.776600 -3.578800
H -2.310400 4.540500 -4.340200
C -1.207600 2.789100 -3.735800
H -0.567300 2.756600 -4.612800
C -1.072400 1.830200 -2.740600
H -0.328500 1.039800 -2.807600
C -2.317100 -2.198400 -1.285600
H -1.259700 -2.338200 -1.069600
C -3.097900 -3.218600 -1.799200
H -2.656700 -4.187900 -2.013200
C -4.451100 -2.970300 -2.014600
H -5.100400 -3.750300 -2.404800
C -4.966100 -1.718400 -1.712800
H -6.020700 -1.496700 -1.854900
C -4.114900 -0.738500 -1.210600
C -4.598400 0.656500 -0.920900
H -4.515900 1.234700 -1.847600

2a

54
Sextet ($S = 5/2$)
C 9.701100 3.515500 1.459900
H 10.766600 3.290700 1.459800
O 4.499400 -0.024300 -0.256600
C 9.272400 4.835500 1.581900
H 10.001600 5.637100 1.670700
C 7.907100 5.120400 1.588000
H 7.564600 6.149100 1.680800
C 6.977600 4.098300 1.469400
Fe 6.542600 0.338300 -0.006200
C 7.404900 2.767400 1.333700
H 5.909100 4.301400 1.460800
O 6.508800 1.802000 1.167500
N 8.771600 0.356700 0.032200
N 7.021000 -1.212300 1.401300
N 7.154100 -1.225100 -1.454100
N 7.014400 1.706100 -1.590500
C 6.249300 -1.682300 2.386300
H 5.253100 -1.254600 2.471800

C 6.697500 -2.667000 3.255800
H 6.042700 -3.029100 4.042500
C 7.985000 -3.164300 3.098100
H 8.366700 -3.930600 3.767900
C 8.789300 -2.667100 2.076800
H 9.804200 -3.025100 1.928900
C 8.268200 -1.689500 1.247900
C 9.005600 -1.107700 0.064300
H 10.073200 -1.353700 0.115300
C 8.377700 -1.705800 -1.176100
C 8.987200 -2.677200 -1.952100
H 9.981800 -3.034800 -1.700100
C 8.301600 -3.161300 -3.062100
H 8.755300 -3.919000 -3.695900
C 7.042400 -2.654400 -3.357400
H 6.481700 -2.998900 -4.221200
C 6.502500 -1.680400 -2.528600
H 5.527200 -1.242700 -2.731000
C 6.086800 2.481500 -2.170200
H 5.048800 2.234000 -1.957500
C 6.424200 3.544200 -2.990100
H 5.643300 4.146900 -3.443700
C 7.772400 3.819900 -3.198800
H 8.074600 4.657400 -3.822700
C 8.730100 3.018200 -2.595400
H 9.791200 3.208200 -2.734000
C 8.317300 1.957000 -1.798300
C 9.302000 1.004100 -1.185400
H 9.537300 0.236600 -1.930400
H 10.243600 1.518400 -0.966000
C 9.250500 1.047900 1.259100
H 8.867500 0.492800 2.120800
H 10.346600 0.998800 1.294900
C 8.791500 2.470900 1.338300
H 4.098100 -0.876400 -0.483500
H 3.811200 0.496700 0.184000

2b

59
Sextet ($S = 5/2$)
F 2.084100 0.593700 -3.518000
C 1.738400 -0.683900 -3.284800
F 0.403300 -0.784600 -3.310100
F 2.183000 -1.040100 -2.072100
S 2.504400 -1.798100 -4.575300
O 3.932700 -1.560700 -4.497000
O 2.014900 -3.132900 -4.287100
O 1.877200 -1.226000 -5.823900
C -0.674900 1.469400 -9.273600
Fe 2.376900 -0.701500 -7.679200
C 0.690200 1.150500 -9.172800
H -1.293200 1.375200 -8.383500
O 1.181300 0.724100 -8.016700
N 3.266800 -0.398100 -9.723000
N 4.155400 0.449200 -7.299000

N 3.886800 -2.337600 -7.957400
N 1.136100 -1.937900 -8.911800
C 4.393900 1.223800 -6.236800
H 3.616300 1.261400 -5.478200
C 5.575700 1.938900 -6.106700
H 5.738700 2.555500 -5.227900
C 6.527000 1.848000 -7.115200
H 7.460700 2.399800 -7.043800
C 6.272200 1.047400 -8.224500
H 6.989100 0.959300 -9.036000
C 5.070600 0.361900 -8.278000
C 4.695600 -0.596400 -9.386400
H 5.350500 -0.454600 -10.255200
C 4.853200 -1.990900 -8.820800
C 5.908600 -2.832000 -9.133700
H 6.670300 -2.515200 -9.840800
C 5.952200 -4.085700 -8.532400
H 6.764000 -4.772300 -8.759100
C 4.940900 -4.452600 -7.653000
H 4.933900 -5.426600 -7.172700
C 3.920600 -3.547900 -7.392900
H 3.104500 -3.794300 -6.716800
C -0.010700 -2.465300 -8.460100
H -0.173200 -2.413500 -7.385300
C -0.942100 -3.036200 -9.309700
H -1.858600 -3.455400 -8.905400
C -0.678600 -3.041300 -10.676400
H -1.395200 -3.465700 -11.375100
C 0.507200 -2.491100 -11.140900
H 0.741200 -2.474800 -12.202000
C 1.404200 -1.949400 -10.227200
C 2.739000 -1.411500 -10.658600
H 3.437500 -2.253100 -10.712900
H 2.672700 -1.001500 -11.672100
C 2.982000 0.978200 -10.205800
H 3.431800 1.675600 -9.492400
H 3.475900 1.131300 -11.174600
C 1.516600 1.259200 -10.319300
C 0.951900 1.665700 -11.523700
H 1.587800 1.745100 -12.404400
C -0.403600 1.972100 -11.618600
H -0.825400 2.284700 -12.570800
C -1.212600 1.873000 -10.486700
H -2.273300 2.107900 -10.552100

2c

55
Doublet ($S = 1/2$)
O 1.222891 0.682576 -2.100426
Fe 1.318046 0.885433 -4.135885
C -0.937337 2.425367 -4.684835
H -2.417071 2.625510 -3.143868
O 0.026238 2.155152 -3.803643
N 1.354450 1.049543 -6.080844
N 2.794796 2.193104 -4.205684

N 2.691459 -0.491708 -4.473522
N -0.070364 -0.461803 -4.450753
C 3.180620 3.112933 -3.314837
H 2.655758 3.130371 -2.365091
C 4.206973 4.002403 -3.597400
H 4.499434 4.731996 -2.848630
C 4.838281 3.942822 -4.833789
H 5.641764 4.632698 -5.077432
C 4.423546 2.996505 -5.765870
H 4.877510 2.925722 -6.749898
C 3.396470 2.142772 -5.413422
C 2.851102 1.032646 -6.268663
H 3.142025 1.124665 -7.319455
C 3.324086 -0.248991 -5.640470
C 4.302851 -1.086096 -6.140731
H 4.785785 -0.857263 -7.086165
C 4.627531 -2.224646 -5.409377
H 5.386572 -2.910181 -5.776378
C 3.962538 -2.480490 -4.215689
H 4.185132 -3.362831 -3.623800
C 2.994554 -1.591392 -3.773370
H 2.445359 -1.751006 -2.850294
C -0.862998 -1.009470 -3.516580
H -0.642640 -0.753320 -2.485109
C -1.909968 -1.850279 -3.851127
H -2.527219 -2.273379 -3.064744
C -2.151017 -2.122773 -5.193179
H -2.973581 -2.769283 -5.487249
C -1.330734 -1.553925 -6.157517
H -1.489349 -1.739967 -7.215917
C -0.291233 -0.729983 -5.754073
C 0.679469 -0.123048 -6.710620
H 1.432877 -0.870853 -6.976699
H 0.187662 0.177115 -7.639696
C 0.734518 2.323109 -6.552065
H 1.343517 3.146541 -6.168392
H 0.784101 2.351172 -7.646212
C -0.674319 2.469009 -6.073608
C -1.720534 2.717348 -6.954139
H -1.518524 2.748979 -8.023533
C -3.015209 2.932471 -6.485125
H -3.823285 3.118235 -7.188462
C -3.266921 2.909400 -5.113835
H -4.275033 3.078153 -4.740821
C -2.238779 2.660854 -4.216342
O 2.458897 0.673094 -1.334901
H 2.260807 -0.037017 -0.695620
H 0.763418 1.480452 -1.767076

2d

61
Doublet ($S = 1/2$)
O 1.185737 -0.182060 -2.341884
Fe 1.336277 0.581739 -3.932126
C -1.090055 2.243602 -4.426653

H -2.678407 1.990838 -2.998207
O -0.060915 2.029074 -3.539419
N 1.393746 1.212310 -5.873945
N 2.724925 1.972513 -3.695695
N 2.737968 -0.583192 -4.553387
N -0.068907 -0.624464 -4.618082
C 3.030553 2.733040 -2.636365
H 2.498525 2.513156 -1.717615
C 3.985717 3.735625 -2.707475
H 4.205705 4.322138 -1.820638
C 4.637642 3.970521 -3.911606
H 5.387962 4.752296 -3.992499
C 4.302971 3.201024 -5.020596
H 4.767653 3.363992 -5.988624
C 3.341079 2.218687 -4.873740
C 2.887670 1.293772 -5.973587
H 3.231888 1.622229 -6.959713
C 3.402209 -0.069647 -5.609076
C 4.442611 -0.747477 -6.215421
H 4.950207 -0.309435 -7.069634
C 4.803184 -1.992016 -5.707849
H 5.614450 -2.552919 -6.163576
C 4.109440 -2.512400 -4.621262
H 4.360330 -3.481707 -4.201533
C 3.071886 -1.783261 -4.062746
H 2.490830 -2.136163 -3.216321
C -0.920460 -1.333395 -3.861036
H -0.754354 -1.284588 -2.788815
C -1.950716 -2.074526 -4.413583
H -2.618733 -2.629043 -3.761557
C -2.110074 -2.080163 -5.794258
H -2.914904 -2.644441 -6.258196
C -1.229556 -1.345000 -6.575952
H -1.324763 -1.316249 -7.657904
C -0.215452 -0.627809 -5.959668
C 0.795017 0.159142 -6.733981
H 1.582003 -0.517210 -7.081496
H 0.346634 0.601010 -7.628552
C 0.729547 2.529172 -6.115920
H 1.246343 3.275731 -5.506261
H 0.860758 2.800784 -7.169498
C -0.722065 2.486564 -5.755157
C -1.729875 2.675985 -6.696781
H -1.457463 2.858338 -7.734814
C -3.069544 2.634090 -6.322225
H -3.846485 2.780081 -7.068673
C -3.410242 2.397540 -4.993275
H -4.455588 2.358813 -4.695384
C -2.420833 2.193436 -4.035435
O 1.854561 0.331561 -1.159863
H 0.402154 1.576276 -0.709251
H 2.819572 0.121281 -1.309791
O 4.505996 -0.157384 -1.483366
H 4.762948 -0.992490 -1.065890
H 5.025019 0.507800 -1.008007
O -0.390663 2.068784 -0.992648

H -0.321121 2.077039 -2.552551
H -0.321092 2.939755 -0.575943

2d dissociates spontaneously. The structure above (of **2d** plus two waters) was used as starting point for linear transit of O-O dissociation. It was obtained by constraining an optimization in O-O bond length starting from **2c** after removing a proton from the proximal oxygen and adding an extra proton to the distal oxygen.

2e

54
Doublet (S = 1/2)
O 1.321743 0.479161 -2.313999
Fe 1.371570 0.871918 -4.050954
C -0.899873 2.461016 -4.730147
H -2.409553 2.643521 -3.212349
O 0.090497 2.321706 -3.864086
N 1.375183 1.053488 -6.076310
N 2.817779 2.179839 -4.162073
N 2.725551 -0.480579 -4.475231
N -0.116153 -0.368940 -4.391247
C 3.199196 3.103137 -3.270933
H 2.690753 3.081318 -2.312271
C 4.197891 4.020737 -3.562048
H 4.487254 4.750235 -2.811258
C 4.808476 3.990669 -4.811412
H 5.589243 4.703659 -5.063536
C 4.397952 3.043462 -5.743233
H 4.833928 2.994523 -6.737116
C 3.397310 2.158678 -5.382611
C 2.863884 1.050942 -6.255624
H 3.171259 1.162077 -7.301224
C 3.353151 -0.228144 -5.642197
C 4.334762 -1.057993 -6.152361
H 4.808023 -0.821380 -7.101055
C 4.679887 -2.192703 -5.425213
H 5.444682 -2.869518 -5.796883
C 4.025680 -2.453364 -4.225430
H 4.264725 -3.331400 -3.632747
C 3.047753 -1.578572 -3.777605
H 2.504164 -1.735599 -2.851555
C -0.961897 -0.835322 -3.462418
H -0.735729 -0.557638 -2.436842
C -2.057584 -1.615210 -3.792270
H -2.717806 -1.971008 -3.006731
C -2.290676 -1.913136 -5.130015
H -3.148680 -2.512715 -5.423487
C -1.415481 -1.424840 -6.091349
H -1.568459 -1.627731 -7.147840
C -0.332477 -0.656344 -5.690452
C 0.685902 -0.128198 -6.654023
H 1.420750 -0.913979 -6.855954
H 0.223266 0.124731 -7.612795
C 0.756613 2.315696 -6.576317
H 1.355203 3.142408 -6.179649
H 0.832747 2.334704 -7.670663
C -0.658278 2.451570 -6.126044

C -1.713653 2.594140 -7.021999
H -1.507756 2.574634 -8.092137
C -3.018360 2.767406 -6.567540
H -3.836636 2.875514 -7.275890
C -3.261630 2.796059 -5.193273
H -4.278056 2.928636 -4.824507
C -2.223131 2.639718 -4.284901
O 2.566352 0.665062 -1.560054
H 2.374882 0.097410 -0.792986

2f

58
Sextet (S = 5/2)
O 1.737900 2.136200 -0.677800
C 1.599400 0.914300 -0.774900
C 1.534700 0.007600 0.427300
H 1.630700 0.578300 1.352500
H 0.584800 -0.537900 0.423800
H 2.334700 -0.738000 0.365200
O 1.507800 0.281500 -1.916200
Fe 1.296100 0.938200 -3.714400
C -1.013900 2.453200 -4.684900
H -2.591600 2.623300 -3.240300
O -0.089700 2.239400 -3.758300
N 1.354400 1.054400 -6.020800
N 2.945000 2.279200 -4.173000
N 2.828300 -0.554600 -4.421600
N -0.157400 -0.526400 -4.363000
C 3.449500 3.209500 -3.356200
H 3.005300 3.260600 -2.363900
C 4.482000 4.048500 -3.756300
H 4.871000 4.791400 -3.066100
C 4.993300 3.917400 -5.041200
H 5.796900 4.563600 -5.385900
C 4.461700 2.951300 -5.890300
H 4.830200 2.824100 -6.904600
C 3.435900 2.150400 -5.417000
C 2.817600 1.024300 -6.216700
H 3.103000 1.106800 -7.273900
C 3.351500 -0.260200 -5.619900
C 4.325000 -1.044300 -6.218600
H 4.722000 -0.777400 -7.194300
C 4.764100 -2.178200 -5.541500
H 5.522300 -2.819100 -5.984700
C 4.219200 -2.483000 -4.299900
H 4.537000 -3.359800 -3.743100
C 3.247500 -1.642300 -3.771600
H 2.785700 -1.826200 -2.803900
C -1.012300 -1.065500 -3.483400
H -0.776600 -0.904100 -2.433600
C -2.130400 -1.777000 -3.883100
H -2.799500 -2.198600 -3.138700
C -2.373100 -1.920200 -5.246200
H -3.250100 -2.458900 -5.597200
C -1.486900 -1.360300 -6.154700

H -1.649500 -1.448100 -7.225800
C -0.377400 -0.670100 -5.678300
C 0.662300 -0.106700 -6.604800
H 1.391000 -0.899300 -6.807100
H 0.211800 0.150200 -7.570600
C 0.746900 2.326800 -6.481800
H 1.333100 3.144800 -6.051200
H 0.832500 2.399500 -7.575300
C -0.682800 2.468800 -6.063500
C -1.693500 2.649100 -7.003200
H -1.434200 2.653400 -8.061300
C -3.019800 2.821800 -6.615600
H -3.794700 2.956900 -7.366700
C -3.342600 2.818700 -5.258600
H -4.376100 2.952700 -4.943700
C -2.354100 2.639300 -4.302000

2g

58
Sextet ($S = 5/2$)
C 2.298800 -0.180200 -0.911700
C 3.544900 -0.121100 0.056100
O 4.639800 0.018100 -0.596500
O 3.388000 -0.214300 1.262300
Cl 0.789600 -0.383200 -0.021500
Cl 2.510200 -1.556000 -2.025400
Cl 2.236500 1.344800 -1.835600
C 6.964600 4.078500 1.473600
Fe 6.486400 0.330200 -0.029600
C 7.391500 2.746300 1.337300
H 5.895200 4.278400 1.472600
O 6.493800 1.781700 1.185100
N 8.768000 0.354800 0.017200
N 7.016200 -1.226700 1.379700
N 7.169600 -1.245200 -1.465400
N 7.013200 1.718900 -1.592500
C 6.247200 -1.703300 2.364100
H 5.244400 -1.287000 2.433500
C 6.706100 -2.678100 3.240600
H 6.053800 -3.045100 4.027400
C 8.000200 -3.159700 3.089900
H 8.390300 -3.917500 3.764800
C 8.799900 -2.656900 2.067900
H 9.819900 -3.002600 1.924400
C 8.269600 -1.688700 1.232500
C 9.012000 -1.103600 0.051200
H 10.080300 -1.348000 0.111900
C 8.393300 -1.716400 -1.186800
C 8.998700 -2.705100 -1.945900
H 9.994700 -3.058800 -1.693500
C 8.301700 -3.215700 -3.036900
H 8.749500 -3.987600 -3.657800
C 7.034600 -2.723600 -3.326000
H 6.462600 -3.096900 -4.170500
C 6.500200 -1.732500 -2.512900

H 5.512200 -1.312200 -2.687500
C 6.085300 2.502600 -2.159100
H 5.049200 2.239700 -1.954600
C 6.422300 3.584200 -2.954500
H 5.641500 4.195500 -3.397500
C 7.770500 3.867200 -3.153700
H 8.072400 4.718800 -3.758600
C 8.728300 3.053900 -2.565800
H 9.789300 3.248700 -2.698300
C 8.314800 1.975700 -1.791000
C 9.296000 1.007300 -1.194800
H 9.512700 0.245000 -1.951000
H 10.246600 1.509700 -0.983500
C 9.244200 1.040400 1.245200
H 8.860400 0.480600 2.103800
H 10.341300 0.995300 1.285900
C 8.780100 2.460600 1.332400
C 9.687500 3.509100 1.446300
H 10.753800 3.286600 1.437300
C 9.257100 4.827800 1.571200
H 9.983900 5.632400 1.655100
C 7.890300 5.105300 1.586200
H 7.543100 6.132500 1.681800

3a

106
Open-shell singlet ($S = 0$)
O -1.183794 0.929507 -9.626502
Fe -0.753438 0.660333 -7.599543
C 1.506877 2.067291 -6.447210
H 2.938931 2.769372 -7.887027
O 0.523356 2.028417 -7.322769
N -0.599201 0.108650 -5.441629
N -2.465929 1.619515 -6.725180
N -2.025717 -1.126402 -7.383836
N 0.890777 -0.729679 -7.592318
C -3.141057 2.673143 -7.195550
H -2.792424 3.095765 -8.134934
C -4.233216 3.199881 -6.519754
H -4.762451 4.052663 -6.933848
C -4.621828 2.619431 -5.319021
H -5.469649 3.012986 -4.764230
C -3.911283 1.528733 -4.826274
H -4.181331 1.052868 -3.887759
C -2.839228 1.056893 -5.562590
C -2.034251 -0.165250 -5.186043
H -2.222341 -0.446038 -4.142608
C -2.470519 -1.271367 -6.123915
C -3.277063 -2.329334 -5.739958
H -3.609791 -2.418135 -4.709547
C -3.630055 -3.272586 -6.700131
H -4.256924 -4.118328 -6.429529
C -3.161215 -3.128389 -7.999773
H -3.407487 -3.848864 -8.773950
C -2.353820 -2.040522 -8.302068

H -1.948972 -1.896863 -9.301723
C 1.707130 -0.883735 -8.644648
H 1.370254 -0.450932 -9.584486
C 2.918044 -1.547232 -8.547017
H 3.549943 -1.652013 -9.423721
C 3.300748 -2.052449 -7.307944
H 4.253184 -2.563180 -7.189690
C 2.455767 -1.890552 -6.219699
H 2.725271 -2.268342 -5.236936
C 1.247284 -1.227232 -6.396550
C 0.256410 -1.084174 -5.277774
H -0.372350 -1.980632 -5.266923
H 0.771315 -1.057879 -4.311562
C -0.073117 1.258106 -4.656199
H -0.764358 2.095392 -4.790994
H -0.069516 0.997050 -3.590016
C 1.299069 1.666296 -5.099251
C 2.372244 1.655362 -4.224925
H 2.206345 1.320587 -3.202390
C 3.672764 1.998227 -4.636463
C 3.854209 2.399149 -5.974122
H 4.842642 2.694788 -6.317991
C 2.796510 2.453808 -6.856130
H 6.876103 0.708166 5.147797
H 8.837401 -0.315031 3.966562
C 7.239358 1.123625 4.211349
C 8.325368 0.552016 3.560537
C 6.613680 2.233631 3.651206
C 8.755553 1.106767 2.363254
H 5.758051 2.702832 4.128592
H 9.596172 0.693468 1.810802
H 5.900324 1.750376 0.572911
C 7.105898 2.732306 2.458185
N 8.158257 2.177976 1.831848
C 6.078329 1.524622 -4.192366
C 5.630731 2.603415 -0.056974
C 4.810878 1.919460 -3.722850
C 7.166521 1.457797 -3.349200
C 4.688401 2.247133 -2.360405
C 5.772160 2.222221 -1.499503
C 7.043335 1.823902 -1.996100
O 8.107710 1.842496 -1.220457
H 6.201454 1.240991 -5.234921
H 4.589494 2.868215 0.168060
H 8.142293 1.144817 -3.713206
H 3.725562 2.577612 -1.974681
C 6.573438 3.974767 1.781810
H 5.594933 4.249351 2.194124
N 6.517694 3.737047 0.318322
Fe 8.656866 3.178131 0.001308
O 10.727598 2.902893 -0.023458
C 7.586770 5.072046 2.033758
C 6.187517 4.950394 -0.457128
H 5.133609 4.941561 -0.754753
N 8.724936 4.934595 1.332413
N 8.313633 4.604241 -1.571630

H 6.444908 6.202761 3.459069
C 7.382549 6.118892 2.916660
C 7.071865 5.108914 -1.660112
H 10.158486 4.329697 -2.464389
C 9.170646 4.768581 -2.589376
H 6.320781 5.832388 0.177844
C 9.692796 5.845644 1.471726
C 6.647504 5.791122 -2.794275
C 8.396177 7.060211 3.070612
H 10.585148 5.707927 0.864690
C 8.822695 5.449212 -3.743070
C 9.567523 6.924522 2.336494
H 5.631958 6.174776 -2.845309
C 7.533550 5.963317 -3.847663
H 8.263992 7.898357 3.749989
H 9.546744 5.560403 -4.544525
H 10.375913 7.644495 2.421115
H 7.220129 6.489416 -4.745963
H -0.781317 1.623279 -10.170370
H 11.169458 2.217458 -0.546958
H 11.338757 3.142969 0.688824
H -2.006041 0.673476 -10.070169

3b

106
Open-shell singlet ($S = 0$)
O -0.771192 0.716102 -9.295045
Fe -0.616110 0.358559 -7.276958
C 1.752241 1.584868 -6.437274
H 3.195663 2.185707 -7.918922
O 0.802850 1.542656 -7.322362
N -0.571820 -0.027071 -5.367035
N -1.979922 1.674438 -6.772389
N -2.058837 -0.966815 -7.279888
N 0.663450 -1.114429 -7.430782
C -2.313828 2.828822 -7.359040
H -1.796560 3.082349 -8.279183
C -3.273118 3.661744 -6.802466
H -3.526851 4.589435 -7.305604
C -3.886452 3.291742 -5.612183
H -4.638882 3.931030 -5.158650
C -3.521212 2.098360 -4.994331
H -3.964538 1.782654 -4.054768
C -2.560023 1.318651 -5.605788
C -2.065746 -0.016932 -5.121148
H -2.316605 -0.211067 -4.074399
C -2.634467 -1.042364 -6.062078
C -3.642499 -1.943355 -5.780473
H -4.078610 -1.982088 -4.786864
C -4.059699 -2.797567 -6.797613
H -4.845345 -3.524225 -6.609868
C -3.455071 -2.721880 -8.046511
H -3.751402 -3.377828 -8.858969
C -2.447434 -1.793398 -8.258075
H -1.933213 -1.704839 -9.210659

C 1.352750 -1.435089 -8.536477
H 1.095151 -0.892745 -9.440821
C 2.342089 -2.402660 -8.516373
H 2.879426 -2.635576 -9.430209
C 2.629984 -3.046159 -7.317640
H 3.409047 -3.802295 -7.270275
C 1.912809 -2.711628 -6.176727
H 2.109269 -3.193920 -5.223530
C 0.925212 -1.743185 -6.266324
C 0.046564 -1.365716 -5.120542
H -0.747192 -2.112360 -5.019473
H 0.597317 -1.359836 -4.176474
C 0.129530 1.028310 -4.583234
H -0.442430 1.955299 -4.684312
H 0.120386 0.747111 -3.525079
C 1.526650 1.273524 -5.058258
C 2.582695 1.322633 -4.182851
H 2.403741 1.086032 -3.137463
C 3.900583 1.622990 -4.617810
C 4.102103 1.913196 -5.993257
H 5.092480 2.173744 -6.353023
C 3.055769 1.933485 -6.871284
H 7.832465 0.246417 4.673139
H 9.793838 -0.143578 3.165402
C 7.987622 0.865296 3.793569
C 9.075757 0.643996 2.959272
C 7.088406 1.885008 3.491366
C 9.249894 1.444849 1.839269
H 6.220243 2.085309 4.112071
H 10.081410 1.314467 1.154251
H 6.089312 1.629669 0.556954
C 7.324555 2.641849 2.361712
N 8.387516 2.426672 1.558447
C 6.333925 1.456944 -4.165859
C 5.694966 2.464821 -0.029223
C 5.008286 1.670789 -3.703366
C 7.404868 1.582492 -3.326492
C 4.819711 1.974705 -2.328704
C 5.882994 2.169641 -1.483298
C 7.212459 2.011021 -1.988994
O 8.261367 2.280384 -1.272809
H 6.506354 1.160733 -5.195830
H 4.633307 2.573246 0.215489
H 8.421995 1.413043 -3.669392
H 3.814372 2.118132 -1.941883
C 6.511893 3.818477 1.898939
H 5.529210 3.877179 2.375813
N 6.415214 3.699199 0.394659
Fe 8.322526 3.627399 0.001340
O 10.353818 3.474132 -0.263342
C 7.359818 5.034981 2.149428
C 5.802636 4.906768 -0.238657
H 4.764594 4.704617 -0.514897
N 8.400236 5.102354 1.291990
N 7.901966 4.924026 -1.404784
H 6.313381 5.887908 3.813771

C 7.163872 5.972876 3.144161
C 6.614133 5.327410 -1.416755
H 9.747867 4.934591 -2.341192
C 8.726653 5.297297 -2.395621
H 5.783939 5.721191 0.491762
C 9.263798 6.121027 1.372428
C 6.112826 6.113157 -2.442155
C 8.072600 7.022373 3.244128
H 10.062495 6.155184 0.637546
C 8.292286 6.091654 -3.441803
C 9.129601 7.098832 2.345274
H 5.068354 6.410957 -2.429966
C 6.963844 6.501725 -3.468103
H 7.946968 7.780930 4.011760
H 8.989572 6.370273 -4.225487
H 9.849809 7.909780 2.385500
H 6.591454 7.115461 -4.283763
H -0.202789 1.441201 -9.601835
H 10.637082 3.009380 -1.065448
H 10.996185 4.180787 -0.107870
H -1.651047 0.899059 -9.658850

3c

106
Open-shell singlet ($S = 0$)
O -0.780208 0.418355 -9.273935
Fe -0.624582 0.252341 -7.300411
C 1.773605 1.562455 -6.554015
H 3.206105 2.108209 -8.074639
O 0.838562 1.423705 -7.423061
N -0.578267 0.037563 -5.349901
N -1.935254 1.648542 -6.886595
N -2.094338 -1.027407 -7.146153
N 0.691334 -1.208903 -7.311217
C -2.222382 2.758941 -7.574489
H -1.694142 2.913630 -8.510047
C -3.157669 3.664281 -7.096735
H -3.383437 4.550286 -7.681213
C -3.781940 3.420049 -5.879740
H -4.512375 4.121357 -5.486375
C -3.455684 2.276271 -5.155335
H -3.905532 2.061336 -4.191025
C -2.524435 1.413096 -5.694198
C -2.068706 0.113401 -5.092247
H -2.304649 0.024508 -4.028723
C -2.671281 -0.975307 -5.929207
C -3.699326 -1.822152 -5.569602
H -4.137676 -1.756859 -4.578650
C -4.133518 -2.755871 -6.506810
H -4.937397 -3.442470 -6.256936
C -3.522316 -2.812668 -7.752765
H -3.830946 -3.533694 -8.502903
C -2.490008 -1.934428 -8.046645
H -1.970010 -1.949317 -8.999840
C 1.406776 -1.606704 -8.373978

H 1.169965 -1.144593 -9.326623
C 2.400649 -2.561971 -8.255417
H 2.957271 -2.861609 -9.137537
C 2.670754 -3.101983 -7.003525
H 3.457062 -3.841494 -6.880007
C 1.927892 -2.683834 -5.907077
H 2.109980 -3.083078 -4.913701
C 0.931260 -1.740324 -6.094430
C 0.020939 -1.288482 -5.002717
H -0.784692 -2.018510 -4.880428
H 0.539729 -1.219516 -4.043652
C 0.152831 1.145114 -4.662342
H -0.407662 2.069380 -4.828987
H 0.139409 0.937621 -3.588172
C 1.550312 1.341049 -5.149312
C 2.602142 1.449686 -4.284753
H 2.424183 1.282147 -3.227141
C 3.925437 1.723440 -4.745463
C 4.119194 1.944401 -6.143420
H 5.105435 2.187911 -6.523257
C 3.074846 1.914079 -7.014077
H 7.932230 -0.056874 4.344178
H 9.852604 -0.320282 2.756913
C 8.058290 0.626905 3.509509
C 9.124626 0.474735 2.631916
C 7.140052 1.654364 3.307261
C 9.260930 1.355000 1.569633
H 6.282573 1.792476 3.958529
H 10.065353 1.268354 0.846018
H 6.109796 1.610973 0.386172
C 7.342545 2.501405 2.237860
N 8.391568 2.356712 1.399526
C 6.362734 1.633244 -4.323539
C 5.709017 2.476415 -0.148995
C 5.026455 1.800657 -3.847582
C 7.430231 1.754710 -3.489487
C 4.835900 2.065072 -2.456721
C 5.893378 2.263007 -1.615539
C 7.231331 2.148752 -2.135254
O 8.263374 2.427275 -1.424403
H 6.539150 1.369511 -5.360622
H 4.647964 2.562797 0.103625
H 8.449954 1.616907 -3.837142
H 3.830804 2.164794 -2.058894
C 6.499916 3.692994 1.878374
H 5.514512 3.683739 2.351159
N 6.404191 3.695151 0.367267
Fe 8.326775 3.690039 -0.031053
O 10.288871 3.751592 -0.338378
C 7.314890 4.905300 2.221296
C 5.776288 4.934406 -0.187182
H 4.737140 4.733025 -0.458183
N 8.354458 5.045498 1.374681
N 7.860950 5.044092 -1.380076
H 6.245852 5.646166 3.922863
C 7.096713 5.783381 3.262841

C 6.567878 5.429133 -1.350005
H 9.705335 5.151707 -2.318178
C 8.676391 5.495077 -2.345120
H 5.761815 5.702349 0.591643
C 9.203198 6.071504 1.505123
C 6.046360 6.270750 -2.318383
C 7.985485 6.843837 3.419683
H 10.005126 6.149697 0.777173
C 8.220580 6.356085 -3.327964
C 9.044243 6.989108 2.532532
H 4.997684 6.550095 -2.279422
C 6.885220 6.742269 -3.319599
H 7.841494 7.558477 4.225050
H 8.909055 6.702878 -4.091772
H 9.749696 7.809045 2.620838
H 6.497058 7.403770 -4.089099
H -0.111942 0.957522 -9.728828
H 10.638973 3.207211 -1.062965
H 10.912139 3.673111 0.401611
H -1.635321 0.618607 -9.687164

3d

106
Open-shell singlet ($S = 0$)
O 2.209646 2.566479 -7.159565
Fe 1.112732 1.540070 -6.156596
C 0.901297 2.791522 -3.556173
H 1.942023 4.618612 -3.058440
O 1.254019 2.880661 -4.763655
N -0.139452 0.334454 -5.108003
N -0.577850 2.227438 -6.859304
N 0.969525 0.158598 -7.503702
N 2.507328 0.431229 -5.317891
C -0.834492 3.435451 -7.372249
H -0.009821 4.140951 -7.390339
C -2.093474 3.744891 -7.865283
H -2.271801 4.730357 -8.284182
C -3.099442 2.787328 -7.816297
H -4.092549 3.011543 -8.195770
C -2.826446 1.535086 -7.273681
H -3.585101 0.760581 -7.212042
C -1.549116 1.292607 -6.807236
C -1.036965 -0.014634 -6.265244
H -1.842472 -0.699158 -5.981344
C -0.147391 -0.579614 -7.341315
C -0.420712 -1.681015 -8.130005
H -1.325029 -2.259291 -7.965301
C 0.495571 -2.021706 -9.121171
H 0.316893 -2.886262 -9.754671
C 1.639255 -1.249709 -9.288511
H 2.371704 -1.484997 -10.054737
C 1.852201 -0.160230 -8.457022
H 2.725636 0.480124 -8.538029
C 3.825764 0.621887 -5.493615
H 4.098515 1.509197 -6.054553

C 4.762757 -0.279788 -5.023495
H 5.818197 -0.096849 -5.204060
C 4.326189 -1.420269 -4.358443
H 5.037467 -2.153269 -3.987252
C 2.963796 -1.615725 -4.183479
H 2.582079 -2.499315 -3.679158
C 2.073928 -0.671221 -4.676803
C 0.592459 -0.845232 -4.570247
H 0.292207 -1.747816 -5.112100
H 0.306009 -1.010860 -3.527284
C -0.886307 1.034908 -4.026883
H -1.550076 1.763359 -4.500832
H -1.518655 0.313028 -3.498542
C -0.009827 1.767959 -3.056607
C -0.133078 1.603772 -1.721673
C 0.623447 2.403893 -0.728440
C 1.312504 3.578535 -1.309195
H 1.739447 4.303827 -0.620829
C 1.425940 3.764000 -2.632316
H 9.871968 0.877596 3.231654
H 9.611085 -0.858011 1.443321
C 9.314872 1.095283 2.324605
C 9.169881 0.128205 1.337269
C 8.737498 2.348972 2.144595
C 8.448047 0.427806 0.191498
H 8.822352 3.131212 2.892949
H 8.306913 -0.285725 -0.614253
H 5.788027 2.115566 1.447288
C 8.039300 2.581768 0.975803
N 7.898822 1.635486 0.024525
C 2.072684 0.288785 -0.694305
C 5.188808 2.837650 0.885770
C 1.648945 1.465295 0.097997
C 3.345011 0.096024 -1.071095
C 2.778434 2.266481 0.628215
C 4.059391 2.098261 0.234337
C 4.360583 1.065501 -0.749859
O 5.472072 0.966175 -1.337532
H 1.309336 -0.434006 -0.971996
H 4.801293 3.568558 1.603787
H 3.654321 -0.763063 -1.657987
H 2.538552 3.029760 1.367281
C 7.409962 3.885254 0.562970
H 7.296421 4.582973 1.398527
N 6.094264 3.526515 -0.075450
Fe 6.868556 2.301748 -1.498179
O 7.627373 1.255322 -2.759658
C 8.285303 4.432372 -0.534366
C 5.415432 4.695830 -0.698090
H 4.448853 4.860095 -0.212348
N 8.216839 3.678463 -1.650526
N 5.768085 3.397226 -2.709610
H 9.141807 6.124051 0.468051
C 9.117209 5.532492 -0.442342
C 5.226216 4.505574 -2.169143
H 6.178128 2.304955 -4.405784

C 5.681686 3.195370 -4.035403
H 6.000106 5.604122 -0.520140
C 8.971955 3.980040 -2.712829
C 4.567119 5.442869 -2.952970
C 9.904435 5.854064 -1.544770
H 8.871389 3.328228 -3.575663
C 5.037361 4.090072 -4.869694
C 9.833127 5.066896 -2.688124
H 4.149234 6.331260 -2.487203
C 4.470456 5.235235 -4.321212
H 10.565413 6.715773 -1.509883
H 5.007636 3.897707 -5.938359
H 10.435254 5.287841 -3.564220
H 3.968126 5.963464 -4.952465
H 1.057171 1.098845 0.950249
H -0.811445 0.845713 -1.332291
H -0.083996 2.770489 0.030027
H 2.405651 3.372177 -6.649834
H 7.081762 0.453466 -2.840909

3e

104
Open-shell singlet ($S = 0$)
O -1.021029 1.341378 -9.446224
Fe -0.750562 0.968858 -7.636671
C 1.313472 2.319849 -6.090420
H 2.779368 3.210522 -7.381625
O 0.351173 2.408523 -6.993132
N -0.659440 0.116359 -5.458689
N -2.607541 1.609877 -6.674721
N -1.877373 -0.975883 -7.620612
N 1.031415 -0.301841 -7.601765
C -3.369785 2.639790 -7.052398
H -3.033061 3.192696 -7.926668
C -4.531317 2.982125 -6.372298
H -5.128429 3.823489 -6.711465
C -4.903075 2.230273 -5.264423
H -5.805538 2.473141 -4.708899
C -4.106004 1.160198 -4.869901
H -4.362234 0.551484 -4.006908
C -2.964566 0.881739 -5.604471
C -2.065622 -0.303881 -5.327701
H -2.293181 -0.728401 -4.340114
C -2.357604 -1.317392 -6.415702
C -3.080025 -2.481940 -6.207349
H -3.447271 -2.729017 -5.214769
C -3.302256 -3.323353 -7.293037
H -3.856335 -4.249577 -7.161329
C -2.800205 -2.970607 -8.540150
H -2.949864 -3.604300 -9.409569
C -2.090071 -1.782803 -8.662589
H -1.673489 -1.457383 -9.613773
C 1.933381 -0.233803 -8.590140
H 1.602485 0.262665 -9.500048
C 3.213256 -0.748457 -8.464115

H 3.913198 -0.671526 -9.291060
C 3.573709 -1.341745 -7.258122
H 4.575808 -1.739392 -7.114460
C 2.639391 -1.413835 -6.235642
H 2.888222 -1.863037 -5.277463
C 1.367770 -0.889381 -6.442168
C 0.291509 -1.004002 -5.398752
H -0.243045 -1.943226 -5.576967
H 0.738646 -1.091442 -4.401365
C -0.278911 1.192052 -4.509998
H -1.022901 1.989746 -4.599872
H -0.316025 0.812134 -3.478647
C 1.084276 1.727112 -4.823082
C 2.141481 1.584732 -3.933467
H 1.959264 1.100855 -2.974099
C 3.444889 1.973049 -4.265796
C 3.655285 2.564368 -5.521459
H 4.657389 2.889817 -5.795631
C 2.612324 2.755541 -6.407392
H 7.050582 1.391262 5.598429
H 8.922776 0.124894 4.509853
C 7.380202 1.640501 4.592824
C 8.416269 0.934916 3.993342
C 6.764272 2.671886 3.891082
C 8.803693 1.282812 2.706052
H 5.949224 3.244549 4.325479
H 9.609700 0.765574 2.190097
H 5.885835 1.752573 0.936247
C 7.212376 2.959239 2.611923
N 8.213211 2.275169 2.034685
C 5.781650 1.218478 -3.840071
C 5.583207 2.534150 0.232643
C 4.575426 1.749919 -3.357157
C 6.878595 1.062455 -3.013856
C 4.511596 2.108716 -2.005575
C 5.613657 2.004571 -1.166991
C 6.829707 1.479245 -1.671809
O 7.923395 1.442775 -0.928670
H 5.850953 0.917595 -4.884097
H 4.566792 2.855929 0.501863
H 7.814422 0.657926 -3.394062
H 3.593958 2.548476 -1.615448
C 6.682818 4.109821 1.783630
H 5.745483 4.486746 2.215813
N 6.529892 3.667061 0.386761
Fe 8.715032 2.926940 0.009488
O 10.557195 2.648326 -0.107825
C 7.750608 5.184279 1.823399
C 6.202537 4.762025 -0.539398
H 5.129366 4.785165 -0.762603
N 8.844350 4.890130 1.105004
N 8.236286 4.171708 -1.724705
H 6.729168 6.564068 3.117158
C 7.635282 6.356190 2.554240
C 6.996749 4.680457 -1.813376
H 10.003800 3.708997 -2.678226

C 9.014178 4.140208 -2.815081
H 6.436306 5.720768 -0.064050
C 9.863259 5.751936 1.081592
C 6.491100 5.154197 -3.019593
C 8.696977 7.255525 2.530179
H 10.722133 5.462227 0.479345
C 8.586484 4.609961 -4.045768
C 9.828885 6.950774 1.783763
H 5.474225 5.535820 -3.065653
C 7.294256 5.116302 -4.149593
H 8.634694 8.188834 3.084489
H 9.249993 4.563849 -4.904423
H 10.675255 7.630069 1.738521
H 6.914558 5.473328 -5.104194
H -0.575704 2.138809 -9.766511
H 10.820607 1.861013 -0.605268

3f

104
Open-shell singlet ($S = 0$)
Fe 0.718930 1.543434 -6.550575
C 0.804299 2.757392 -3.881559
O 1.573482 2.441634 -7.622711
H 2.009712 4.516935 -3.539637
O 0.942447 2.881805 -5.128070
N -0.388559 0.303329 -5.271536
N -1.071233 2.165543 -7.034941
N 0.415187 0.057301 -7.774763
N 2.234620 0.549148 -5.778630
C -1.407609 3.356638 -7.537438
H -0.593928 4.055354 -7.707529
C -2.731080 3.653426 -7.828285
H -2.978548 4.625723 -8.242944
C -3.711121 2.699020 -7.584952
H -4.754013 2.913220 -7.801982
C -3.349637 1.462622 -7.056418
H -4.086779 0.693467 -6.846340
C -2.013021 1.230488 -6.795865
C -1.423552 -0.064871 -6.292600
H -2.186890 -0.742121 -5.896724
C -0.680508 -0.658615 -7.457647
C -1.054980 -1.774081 -8.182570
H -1.939117 -2.336016 -7.897172
C -0.268094 -2.147109 -9.268424
H -0.532718 -3.020792 -9.857635
C 0.857130 -1.396937 -9.587794
H 1.491138 -1.657785 -10.429468
C 1.179355 -0.291358 -8.815418
H 2.041581 0.337366 -9.014648
C 3.518415 0.864275 -6.011330
H 3.680077 1.735995 -6.639027
C 4.554062 0.106792 -5.495963
H 5.580747 0.381090 -5.718927
C 4.250660 -1.004612 -4.717274
H 5.043423 -1.624241 -4.306037

C 2.921012 -1.316149 -4.471335
H 2.646400 -2.177382 -3.868167
C 1.924789 -0.519648 -5.019514
C 0.467934 -0.833043 -4.846934
H 0.225351 -1.720406 -5.440523
H 0.254392 -1.100159 -3.807598
C -1.004132 0.988699 -4.108065
H -1.730375 1.709853 -4.495332
H -1.556129 0.259426 -3.504565
C -0.021393 1.732121 -3.258442
C 0.031524 1.571023 -1.918970
C 0.904400 2.376018 -1.034754
C 1.593795 3.493478 -1.717518
H 2.173499 4.178993 -1.103630
C 1.513461 3.689554 -3.041677
H 9.779480 0.952267 3.839526
H 9.824841 -0.758732 2.010100
C 9.330331 1.162676 2.872815
C 9.355995 0.209207 1.862570
C 8.720822 2.392799 2.640830
C 8.774913 0.502352 0.637556
H 8.674698 3.160945 3.406915
H 8.767405 -0.195356 -0.194499
H 5.856053 2.112353 1.613642
C 8.164143 2.620533 1.397133
N 8.198223 1.688330 0.423380
C 2.437695 0.328906 -1.050439
C 5.315261 2.834917 0.994630
C 1.912010 1.439928 -0.225950
C 3.749396 0.139945 -1.254661
C 2.957424 2.244969 0.444993
C 4.278421 2.091158 0.211425
C 4.716197 1.074920 -0.736251
O 5.904580 0.961313 -1.139998
H 1.717438 -0.357469 -1.489807
H 4.840505 3.555206 1.670206
H 4.134049 -0.682038 -1.850581
H 2.619389 2.995690 1.158535
C 7.529508 3.911186 0.937912
H 7.302253 4.581361 1.772916
N 6.312079 3.535152 0.147057
Fe 7.332466 2.311400 -1.217121
O 8.202619 1.425974 -2.287353
C 8.499043 4.519675 -0.037505
C 5.700537 4.669925 -0.590398
H 4.725998 4.918733 -0.159553
N 8.580646 3.807167 -1.177026
N 6.248511 3.308488 -2.526627
H 9.178883 6.203354 1.099368
C 9.274248 5.643898 0.173755
C 5.564783 4.368609 -2.053936
H 6.796085 2.142737 -4.132589
C 6.209694 3.007028 -3.834006
H 6.318609 5.565627 -0.469719
C 9.428604 4.166418 -2.146404
C 4.814770 5.168860 -2.905345

C 10.159680 6.029503 -0.828375
H 9.444861 3.538201 -3.031621
C 5.484235 3.769206 -4.731302
C 10.238561 5.281700 -1.996779
H 4.277365 6.023060 -2.502324
C 4.779301 4.871389 -4.260282
H 10.780970 6.911000 -0.696183
H 5.489295 3.506076 -5.784750
H 10.920154 5.551947 -2.797348
H 4.208835 5.495371 -4.943582
H 1.287316 0.985602 0.561871
H -0.589786 0.813501 -1.442399
H 0.264184 2.823626 -0.255375

3f

106
Open-shell singlet ($S = 0$)
O 1.250399 2.498490 -7.959281
Fe 0.520008 1.527682 -6.710090
C 0.798387 2.745670 -4.090618
H 2.099251 4.457930 -3.879283
O 0.799542 2.862111 -5.352405
N -0.438781 0.286768 -5.401345
N -1.321070 2.089568 -7.061943
N 0.232911 -0.015462 -7.884649
N 2.129130 0.640387 -5.993201
C -1.717384 3.292924 -7.485027
H -0.944531 4.033478 -7.665458
C -3.066363 3.548873 -7.683990
H -3.368222 4.528853 -8.039471
C -3.996497 2.551093 -7.424794
H -5.056788 2.737735 -7.569279
C -3.567037 1.308165 -6.965271
H -4.264155 0.509683 -6.731465
C -2.212667 1.113511 -6.795972
C -1.541780 -0.149784 -6.337828
H -2.229150 -0.848917 -5.854958
C -0.844249 -0.740541 -7.525676
C -1.206109 -1.882632 -8.209976
H -2.070856 -2.455222 -7.889709
C -0.422585 -2.274680 -9.292209
H -0.678297 -3.170588 -9.850426
C 0.694651 -1.526388 -9.638808
H 1.333576 -1.808165 -10.469449
C 1.010099 -0.393048 -8.905838
H 1.881333 0.216044 -9.125257
C 3.375937 1.060060 -6.256893
H 3.469182 1.918361 -6.915786
C 4.471075 0.406843 -5.720758
H 5.469707 0.747859 -5.975248
C 4.264323 -0.685663 -4.886082
H 5.108902 -1.219568 -4.458200
C 2.968007 -1.096592 -4.607716
H 2.769432 -1.948168 -3.963318
C 1.907871 -0.415150 -5.186251

C 0.482735 -0.822780 -5.008440
H 0.279983 -1.698096 -5.632525
H 0.279160 -1.121583 -3.977306
C -0.995881 0.975237 -4.199530
H -1.760696 1.677813 -4.539927
H -1.487787 0.221374 -3.577116
C 0.024772 1.727271 -3.409425
C 0.156729 1.583399 -2.072500
C 1.075795 2.408361 -1.262384
C 1.775170 3.471232 -2.013701
H 2.431881 4.135721 -1.456836
C 1.608750 3.657385 -3.334179
H 9.664186 0.973111 4.109921
H 9.662857 -0.773659 2.313353
C 9.251956 1.188697 3.128438
C 9.247806 0.213945 2.139371
C 8.714328 2.445228 2.861249
C 8.711250 0.504207 0.893850
H 8.681092 3.228348 3.612055
H 8.686472 -0.219672 0.086826
H 5.890393 2.116088 1.658135
C 8.203670 2.674295 1.601257
N 8.218658 1.722366 0.647366
C 2.621371 0.385760 -1.210937
C 5.393225 2.846708 1.015237
C 2.059596 1.496452 -0.414307
C 3.942720 0.180525 -1.335582
C 3.061488 2.306232 0.308882
C 4.393497 2.127266 0.165228
C 4.869445 1.103615 -0.745956
O 6.090771 0.987097 -1.064656
H 1.922618 -0.288655 -1.700657
H 4.895447 3.575043 1.662264
H 4.357005 -0.642374 -1.910364
H 2.685790 3.068178 0.990217
C 7.620114 3.956427 1.087969
H 7.330732 4.648405 1.882842
N 6.444647 3.559339 0.230073
Fe 7.468994 2.338587 -1.050002
O 8.440098 1.386078 -2.124685
C 8.635965 4.535210 0.144085
C 5.862204 4.722355 -0.501176
H 4.903014 4.998594 -0.055860
N 8.738749 3.811771 -0.988632
N 6.406776 3.335390 -2.390163
H 9.311424 6.218978 1.279854
C 9.422803 5.646676 0.364258
C 5.720352 4.406217 -1.948486
H 6.965744 2.135125 -3.987411
C 6.382178 3.000641 -3.692866
H 6.518827 5.590252 -0.385645
C 9.605088 4.148196 -1.948414
C 4.974635 5.186352 -2.820111
C 10.337776 6.006209 -0.622335
H 9.632736 3.519510 -2.832861
C 5.666247 3.749226 -4.607791

C 10.430868 5.250450 -1.783671
H 4.434695 6.047546 -2.437050
C 4.955372 4.860133 -4.167803
H 10.968965 6.879267 -0.483502
H 5.689022 3.467486 -5.655956
H 11.135004 5.502037 -2.570250
H 4.395278 5.470883 -4.870803
H 1.411718 1.035727 0.353588
H -0.434993 0.836143 -1.546440
H 0.456675 2.923163 -0.504220
H 8.061235 0.512933 -2.351782
H 0.961155 2.397252 -8.884397

3h

104
Open-shell singlet ($S = 0$)
O -0.825843 0.635125 -9.104322
Fe -0.644524 0.393968 -7.332561
C 1.639296 1.762299 -6.439578
H 3.063024 2.439925 -7.904429
O 0.696327 1.704591 -7.336064
N -0.603005 0.030823 -5.338558
N -2.086098 1.621290 -6.791426
N -2.020918 -1.003889 -7.284125
N 0.771599 -0.979127 -7.370563
C -2.490895 2.724941 -7.425404
H -1.990546 2.960081 -8.359442
C -3.509657 3.505956 -6.899629
H -3.827875 4.394086 -7.436528
C -4.101671 3.134774 -5.698704
H -4.897114 3.737123 -5.268550
C -3.666881 1.985532 -5.043694
H -4.099408 1.666168 -4.100363
C -2.652578 1.251245 -5.625725
C -2.087613 -0.047894 -5.119853
H -2.352624 -0.250025 -4.077869
C -2.588173 -1.108962 -6.065459
C -3.544076 -2.065776 -5.784213
H -3.974785 -2.131152 -4.789550
C -3.919684 -2.936416 -6.804079
H -4.663773 -3.705609 -6.615980
C -3.332006 -2.817166 -8.057261
H -3.604628 -3.477776 -8.874399
C -2.374765 -1.835271 -8.268654
H -1.879341 -1.690935 -9.224105
C 1.524491 -1.254313 -8.446986
H 1.251662 -0.749463 -9.367735
C 2.590335 -2.133143 -8.376321
H 3.176112 -2.330500 -9.268606
C 2.890582 -2.732936 -7.158391
H 3.730606 -3.416759 -7.071297
C 2.105162 -2.446708 -6.050708
H 2.306083 -2.896785 -5.082754
C 1.040979 -1.568345 -6.188224
C 0.093636 -1.258219 -5.077348

H -0.647852 -2.060752 -5.012807
H 0.605940 -1.224084 -4.111855
C 0.039549 1.138776 -4.582882
H -0.576018 2.034050 -4.711105
H 0.042844 0.889599 -3.516106
C 1.426309 1.428005 -5.066824
C 2.487920 1.477703 -4.193445
H 2.316793 1.218492 -3.151441
C 3.795833 1.800258 -4.625900
C 3.983956 2.137909 -5.988078
H 4.969334 2.425010 -6.343883
C 2.931207 2.159158 -6.862694
H 7.665074 0.240930 4.692686
H 9.592333 -0.272420 3.177285
C 7.844472 0.838149 3.802884
C 8.913122 0.547056 2.964166
C 6.998758 1.900529 3.495387
C 9.118042 1.319740 1.830428
H 6.149108 2.156116 4.121188
H 9.935487 1.143770 1.138364
H 5.985903 1.665544 0.551870
C 7.263910 2.631701 2.354705
N 8.303914 2.340297 1.548591
C 6.221637 1.523568 -4.182425
C 5.634102 2.518584 -0.035736
C 4.914680 1.802478 -3.714893
C 7.299196 1.568553 -3.339145
C 4.747696 2.105367 -2.342700
C 5.822006 2.222899 -1.491498
C 7.137276 1.980620 -1.993960
O 8.209012 2.147545 -1.272742
H 6.375684 1.239184 -5.219428
H 4.574721 2.669777 0.198775
H 8.304062 1.344398 -3.687615
H 3.752193 2.306550 -1.954546
C 6.519708 3.850874 1.884134
H 5.550169 3.974935 2.375758
N 6.402976 3.718292 0.392189
Fe 8.379168 3.533878 -0.017404
O 10.159818 3.463821 -0.245711
C 7.447932 5.014253 2.123014
C 5.868241 4.943310 -0.261485
H 4.815873 4.804754 -0.524284
N 8.503976 4.996089 1.284845
N 7.954873 4.820404 -1.453419
H 6.442049 5.970880 3.756174
C 7.305914 5.981903 3.098413
C 6.690080 5.287450 -1.458401
H 9.797310 4.733463 -2.385544
C 8.792362 5.136910 -2.453183
H 5.910119 5.776790 0.446620
C 9.450465 5.935564 1.369771
C 6.220351 6.076960 -2.496374
C 8.289141 6.962959 3.196904
H 10.271132 5.860131 0.662749
C 8.386291 5.931606 -3.510439

C 9.369789 6.939019 2.324360
H 5.192153 6.426875 -2.479489
C 7.078282 6.402067 -3.537555
H 8.205455 7.743404 3.948312
H 9.091469 6.164481 -4.302139
H 10.152683 7.689444 2.373244
H 6.728255 7.015381 -4.363520
H -0.119454 1.233030 -9.408235
H 10.352024 2.850547 -0.977691

4a

53
Triplet ($S = 1$)
O 1.443068 0.694096 -2.307386
Fe 1.312988 0.914581 -4.084297
C -0.933620 2.461732 -4.698384
H -2.395463 2.729804 -3.147808
O 0.010390 2.196609 -3.806597
N 1.353180 1.059154 -6.108055
N 2.787718 2.221197 -4.250908
N 2.715001 -0.442999 -4.446390
N -0.080373 -0.431155 -4.440045
C 3.179914 3.113297 -3.338249
H 2.648211 3.108024 -2.391995
C 4.226905 3.982184 -3.609035
H 4.536874 4.692206 -2.848738
C 4.857407 3.924141 -4.845730
H 5.675805 4.600129 -5.077935
C 4.433502 2.994455 -5.791431
H 4.896399 2.921253 -6.770848
C 3.391022 2.154619 -5.453760
C 2.842989 1.032880 -6.292185
H 3.139474 1.101683 -7.342837
C 3.321117 -0.233762 -5.631778
C 4.298366 -1.082172 -6.114425
H 4.763892 -0.887258 -7.075889
C 4.649996 -2.183965 -5.339051
H 5.410238 -2.875776 -5.691464
C 4.018465 -2.392091 -4.119240
H 4.271220 -3.239906 -3.490185
C 3.044826 -1.498088 -3.696140
H 2.518642 -1.604732 -2.752131
C -0.856606 -0.977856 -3.491371
H -0.620318 -0.718368 -2.464898
C -1.900867 -1.824368 -3.816328
H -2.507806 -2.247935 -3.022338
C -2.153457 -2.100979 -5.155366
H -2.976727 -2.750641 -5.439662
C -1.342416 -1.536103 -6.129000
H -1.505093 -1.730986 -7.185055
C -0.301123 -0.707110 -5.740870
C 0.672266 -0.121542 -6.707344
H 1.420166 -0.880621 -6.957567
H 0.182721 0.160086 -7.643511
C 0.728320 2.322513 -6.586172

H 1.337564 3.155248 -6.222367
H 0.762537 2.340665 -7.681059
C -0.675481 2.476667 -6.092778
C -1.721854 2.737505 -6.965370
H -1.528699 2.757974 -8.036186
C -3.009862 2.984980 -6.488553
H -3.817526 3.177595 -7.190136
C -3.256619 2.991575 -5.114901
H -4.258697 3.187540 -4.740325
C -2.229232 2.741392 -4.222321
H 0.723019 1.191440 -1.879363

4b

52
Triplet (S = 1)
O 1.441501 0.638173 -2.382787
Fe 1.345150 0.908145 -4.004187
C -0.927156 2.469280 -4.758104
H -2.450698 2.592315 -3.248898
O 0.059041 2.365234 -3.878223
N 1.358150 1.090054 -6.101006
N 2.806622 2.209953 -4.174819
N 2.709537 -0.463959 -4.473225
N -0.115606 -0.351443 -4.386275
C 3.221648 3.085763 -3.255682
H 2.704438 3.057294 -2.300884
C 4.261326 3.964300 -3.523740
H 4.583599 4.660366 -2.755055
C 4.868442 3.935857 -4.774091
H 5.680262 4.619330 -5.008776
C 4.425369 3.024689 -5.728021
H 4.869584 2.973861 -6.717920
C 3.389298 2.172957 -5.390077
C 2.840652 1.071430 -6.265049
H 3.161370 1.176242 -7.307365
C 3.320409 -0.214559 -5.646936
C 4.290863 -1.053394 -6.164010
H 4.755618 -0.825096 -7.118789
C 4.637335 -2.186332 -5.433895
H 5.392915 -2.869577 -5.812636
C 4.003195 -2.435726 -4.221986
H 4.249243 -3.309238 -3.625671
C 3.036224 -1.551154 -3.766870
H 2.508190 -1.686900 -2.827400
C -0.935030 -0.839807 -3.444487
H -0.695180 -0.557567 -2.423575
C -2.015524 -1.642005 -3.766911
H -2.656965 -2.018722 -2.975808
C -2.258985 -1.935412 -5.104317
H -3.107492 -2.551541 -5.390695
C -1.407158 -1.424918 -6.073865
H -1.567623 -1.627780 -7.129193
C -0.334258 -0.635162 -5.685229
C 0.662491 -0.091156 -6.664241
H 1.393361 -0.874575 -6.889679

H 0.175291 0.161593 -7.610931
C 0.744889 2.352595 -6.597520
H 1.336631 3.179089 -6.189753
H 0.824820 2.383946 -7.691556
C -0.673992 2.473552 -6.151426
C -1.726835 2.597053 -7.053028
H -1.513991 2.588599 -8.121824
C -3.038437 2.734795 -6.606139
H -3.853707 2.828528 -7.319933
C -3.293686 2.743433 -5.234026
H -4.315862 2.845540 -4.871958
C -2.257804 2.603686 -4.320209

5

52
Quartet ($S = 3/2$)
O 1.436480 0.641874 -2.406376
Fe 1.299461 0.880999 -4.025034
C -0.964197 2.454381 -4.763209
H -2.443080 2.751644 -3.228463
O -0.070823 2.133598 -3.856102
N 1.330556 1.041645 -6.114204
N 2.756219 2.210107 -4.207911
N 2.695943 -0.477550 -4.463949
N -0.081032 -0.473980 -4.399957
C 3.142159 3.100603 -3.292211
H 2.617363 3.078392 -2.341802
C 4.171386 3.990399 -3.565766
H 4.475292 4.701173 -2.803724
C 4.791760 3.949004 -4.807499
H 5.597685 4.638726 -5.042987
C 4.374821 3.017569 -5.755337
H 4.831485 2.958313 -6.738632
C 3.349013 2.157791 -5.416646
C 2.816727 1.037360 -6.275377
H 3.130814 1.129404 -7.319574
C 3.307854 -0.232889 -5.636658
C 4.300477 -1.060104 -6.124937
H 4.773488 -0.839688 -7.076999
C 4.657370 -2.172668 -5.367883
H 5.430827 -2.846991 -5.725252
C 4.016180 -2.414687 -4.159478
H 4.272195 -3.271579 -3.544369
C 3.028820 -1.541175 -3.727934
H 2.496233 -1.669994 -2.790613
C -0.853544 -1.013452 -3.445198
H -0.609932 -0.739890 -2.423119
C -1.901143 -1.857032 -3.765785
H -2.507564 -2.278626 -2.970307
C -2.159819 -2.130739 -5.104881
H -2.986100 -2.778092 -5.385849
C -1.355248 -1.564840 -6.082957
H -1.527133 -1.756664 -7.138141
C -0.307095 -0.739004 -5.702246
C 0.656587 -0.153223 -6.685178

H 1.407259 -0.910716 -6.932349
H 0.151926 0.101792 -7.621182
C 0.721250 2.293654 -6.628239
H 1.330362 3.130994 -6.272813
H 0.768789 2.293868 -7.723074
C -0.683124 2.480394 -6.157682
C -1.706341 2.783383 -7.038508
H -1.500767 2.808428 -8.106574
C -2.993302 3.071349 -6.573807
H -3.782933 3.298682 -7.285292
C -3.263946 3.073807 -5.202553
H -4.264899 3.300637 -4.843763
C -2.263286 2.775242 -4.300298
